# Deep learning on tertiary lymphoid structures in hematoxylin-eosin predicts cancer prognosis and immunotherapy response

**DOI:** 10.1038/s41698-024-00579-w

**Published:** 2024-03-22

**Authors:** Ziqiang Chen, Xiaobing Wang, Zelin Jin, Bosen Li, Dongxian Jiang, Yanqiu Wang, Mengping Jiang, Dandan Zhang, Pei Yuan, Yahui Zhao, Feiyue Feng, Yicheng Lin, Liping Jiang, Chenxi Wang, Weida Meng, Wenjing Ye, Jie Wang, Wenqing Qiu, Houbao Liu, Dan Huang, Yingyong Hou, Xuefei Wang, Yuchen Jiao, Jianming Ying, Zhihua Liu, Yun Liu

**Affiliations:** 1 MOE Key Laboratory of Metabolism and Molecular Medicine, Department of Biochemistry and Molecular Biology, School of Basic Medical Sciences and Shanghai Xuhui Central Hospital, Fudan University, Shanghai, China; 2 State Key Laboratory of Medical Neurobiology and MOE Frontiers Center for Brain Science, Institutes of Brain Science, Fudan University, Shanghai, China; 3 State Key Laboratory of Molecular Oncology, National Cancer Center, National Clinical Research Center for Cancer, Cancer Hospital, Chinese Academy of Medical Sciences and Peking Union Medical College, Beijing, China; 4 Department of General Surgery/Gastric Cancer Center, Zhongshan Hospital, Fudan University, Shanghai, China; 5 Department of Pathology, Zhongshan Hospital, Fudan University, Shanghai, China; 6 Departments of Pathology, International Peace Maternity and Child Health Hospital Affiliated to Shanghai Jiao Tong University School of Medicine, Shanghai, China; 7 Department of Pathology, National Cancer Center/National Clinical Research Center for Cancer/Cancer Hospital, Chinese Academy of Medical Sciences and Peking Union Medical College, Beijing, China; 8 Thoracic Surgery Department, National Cancer Center/National Clinical Research Center for Cancer/Cancer Hospital, Chinese Academy of Medical Sciences and Peking Union Medical College, Beijing, China; 9 Division of Rheumatology and Immunology, Huashan Hospital, Fudan University, Shanghai, China; 10 Departments of Thoracic Surgery, Fudan University Shanghai Cancer Center, Shanghai, China; 11 Shanghai Xuhui Central Hospital, Shanghai, China; 12 Department of General Surgery/Biliary Tract Disease Center, Zhongshan Hospital, Fudan University, Shanghai, China; 13 Department of Pathology, Fudan University Shanghai Cancer Center, Shanghai, China; Department of Oncology, Shanghai Medical College, Fudan University, Shanghai, China; Institute of Pathology, Fudan University, Shanghai, China; 14 Department of General Surgery, Zhongshan Hospital (Xiamen), Fudan University, Shanghai, China

**Keywords:** Prognostic markers, Tumour biomarkers

## Abstract

Tertiary lymphoid structures (TLSs) have been associated with favorable immunotherapy responses and prognosis in various cancers. Despite their significance, their quantification using multiplex immunohistochemistry (mIHC) staining of T and B lymphocytes remains labor-intensive, limiting its clinical utility. To address this challenge, we curated a dataset from matched mIHC and H&E whole-slide images (WSIs) and developed a deep learning model for automated segmentation of TLSs. The model achieved Dice coefficients of 0.91 on the internal test set and 0.866 on the external validation set, along with intersection over union (IoU) scores of 0.819 and 0.787, respectively. The TLS ratio, defined as the segmented TLS area over the total tissue area, correlated with B lymphocyte levels and the expression of *CXCL13*, a chemokine associated with TLS formation, in 6140 patients spanning 16 tumor types from The Cancer Genome Atlas (TCGA). The prognostic models for overall survival indicated that the inclusion of the TLS ratio with TNM staging significantly enhanced the models’ discriminative ability, outperforming the traditional models that solely incorporated TNM staging, in 10 out of 15 TCGA tumor types. Furthermore, when applied to biopsied treatment-naïve tumor samples, higher TLS ratios predicted a positive immunotherapy response across multiple cohorts, including specific therapies for esophageal squamous cell carcinoma, non-small cell lung cancer, and stomach adenocarcinoma. In conclusion, our deep learning-based approach offers an automated and reproducible method for TLS segmentation and quantification, highlighting its potential in predicting immunotherapy response and informing cancer prognosis.

## Introduction

Tertiary lymphoid structures (TLSs) are organized aggregation of immune cells resembling secondary lymphoid organs^1–3^. While the mechanisms governing TLS formation in tumor microenvironment remain unclear, its presence associates with a positive immunotherapy response in multiple cancers^2,4–7^. A recent clinical trial revealed that the presence of TLS in advanced soft-tissue sarcomas predicts a favorable response to pembrolizumab treatment^8^, underscored its potential as a valuable biomarker for predicting clinical efficacy of immunotherapy. Moreover, several meta-analyses demonstrated the associations between the presence of TLS and prolonged overall survival in gastrointestinal cancers^9^ and digestive system cancers^10^, further highlighting the clinical value of TLS across multiple cancer types.

Currently, the gold standard to segment and quantify TLS is based on pathological characteristics using multiplex immunohistochemistry (mIHC) staining on T and B lymphocytes^11,12^. However, mIHC is resource intensive and not widely available, limiting its clinical utility. While experienced pathologists can potentially identify TLSs on hematoxylin and eosin (H&E)-stained whole-slide images (WSIs)^13,14^, but to our knowledge, the sensitivity and accuracy of this approach to segment TLSs based on H&E staining alone against the results established by mIHC are not systematically evaluated.

With the rise of deep learning, automated histopathological feature extraction has become feasible for a range of tasks, including cancer grading^15,16^, diagnosis^17–19^, prognosis^20–22^, and predicting immunotherapy response^23,24^, molecular expression^25,26^, and genetic alterations^27,28^. Some algorithms can even achieve diagnostic accuracy rivaling pathologists^29,30^. In this work, we curated a dataset from matched mIHC and H&E WSIs and developed a deep-learning approach that segments and calculates the TLS ratio (defined as the segmented TLS area divided by the tissue area) from H&E WSIs. Subsequently, we validated the accuracy of our approach in The Cancer Genome Atlas (TCGA), and evaluated the associations between TLS ratios and overall survival across multiple cancer types. Finally, the TLS ratio was assessed for predicting an immunotherapy response in various cohorts.

## Results

### Data collection and development of the TLS segmentation model

**Fig. 1:**
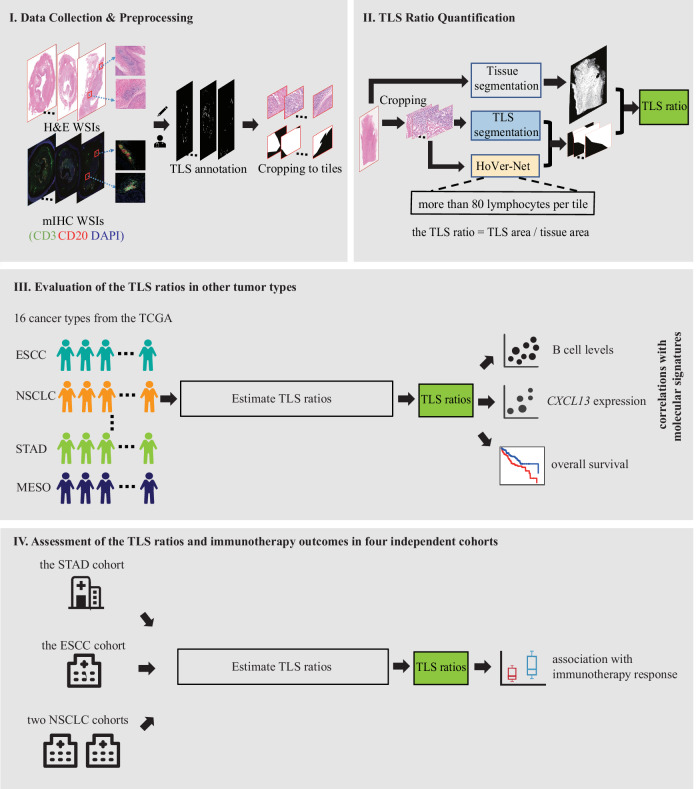
Overview of the study design and methodology. (I) We collected data from 65 patients diagnosed with either ESCC or NSCLC to obtain mIHC and H&E WSIs. TLSs on the H&E WSIs were segmented based on the mIHC WSIs and were further cropped into 22,497 tiles. (II) A deep learning approach was employed to automatically segment TLSs and quantify the TLS ratio, which is calculated by dividing the segmented TLS area by the tissue area. (III) Sixteen cancer cohorts from the TCGA were used to evaluate potential correlations between the TLS ratios, molecular signatures (B cell levels and *CXCL13* expression), and prognostic outcomes. (IV) We evaluated the associations between the TLS ratios and immunotherapy responses in one ESCC cohort, two independent NSCLC cohorts, and one STAD cohort. TCGA, The Cancer Genome Atlas, ESCC, esophageal squamous cell carcinoma, NSCLC, non-small cell lung cancer, STAD, stomach adenocarcinoma.

The overall study design is illustrated in Fig. 1. First, we generated a rigorously curated dataset based on matched mIHC and H&E WSIs (part I of Fig. 1), all at a magnification of 20× (0.5 ${\upmu}{\mathrm{m}}{/}$pixel), from 60 esophageal squamous cell carcinoma (ESCC) patients and 5 non-small cell lung cancer (NSCLC) patients (Supplementary Table 1). TLSs were identified based on CD3 and CD20 staining and subsequently used as ground truth for the segmentation of TLSs on consecutive H&E-stained slides from the same individuals. The H&E WSIs and their TLS segmentations were cropped into 22,497 equally sized tiles (512 × 512 pixels, 256 mm × 256 mm) (Supplementary Fig. 1) and randomly split into internal training, validation and test sets in a ratio of 7:1:2 (Supplementary Table 2).

These tiles were then used to train a model to segment TLSs on H&E WSIs. By using a modified encoder-decoder model based on EfficientNet-b0^31^, we achieved a strong segmentation performance with a Dice coefficient of 0.91 (95% confidence interval [CI]: 0.902–0.918) and an intersection over union (IoU) of 0.819 (95% CI: 0.811–0.827) on the internal test set. Moreover, the model showed excellent ability to discriminate TLSs, with areas under the curve (AUCs) for the receiver operator characteristic (ROC) curves (Supplementary Fig. 2a–c) reaching 0.981 (95% CI: 0.892–0.999), 0.965 (95% CI: 0.873–0.998), and 0.966 (95% CI: 0.869–0.989) for the internal training, validation, and test sets, respectively. Examples of TLS segmentation on the holdout internal test set were illustrated in Supplementary Fig. 3. Evaluation of the model’s predictive accuracy was extended by assessing the linear correlations between the predicted and observed TLS area for each tile. These analyses revealed strong correlations across all three internal data sets (all rho >0.89), with highly significant *P* values (all *P* values < 0.0001) (Supplementary Fig. 4a–c). Additionally, our analysis did not reveal any significant prediction bias across different samples, as the IoUs for individual slides were consistently above 0.7 (Supplementary Fig. 4d).

To further validate the accuracy of our model, we assembled an external validation set comprised of five ESCC and ten NSCLC samples obtained from the TCGA. From these H&E-stained WSIs, we generated a total of 667 tiles for TLS segmentation. The performance of our model on this external validation set remained robust, as evidenced by a Dice coefficient of 0.866 (95% CI: 0.855–0.877), an IoU of 0.787 (95% CI: 0.773–0.802), and an AUC of 0.934 (95% CI: 0.838–0.968) (Supplementary Fig. 2d). A significant linear correlation between the predicted and actual TLS areas per tile was observed (rho = 0.79, *P* value < 0.0001) (Supplementary Fig. 4e). Moreover, the IoUs for individual slides were consistently above 0.6 (Supplementary Fig. 4f). Collectively, these results underscore the robustness and reliability of our deep learning model in TLS segmentation.

### Deep learning pipeline for TLS ratio calculation

After TLS segmentation, we employed a deep-learning pipeline to calculate the TLS ratio for each H&E WSI. As illustrated in the part II of Fig. 1, the pipeline comprised three distinct branches and comprehensive details of these branches were provided in the Methods section. Briefly, these branches were designed to determine the tissue area, segmented TLS area, and lymphocyte count, respectively. The branch to determine the tissue area employed the OTSU method from the OpenCV Python package^32^, which segment the tissue region from the non-tissue background. The branch to determine lymphocyte count, specifically designed to exclude small-sized TLSs, utilized the publicly available deep learning model HoVer-Net^33^. This model is broadly used for segmenting different cell types, particularly lymphocytes, from H&E WSIs^33^. Tiles with a lymphocyte count exceeding 80 within the segmented TLSs were retained for the TLS ratio calculation.

### Estimated TLS ratios correlate with B lymphocyte levels and *CXCL13* expression across various TCGA tumor types

**Fig. 2:**
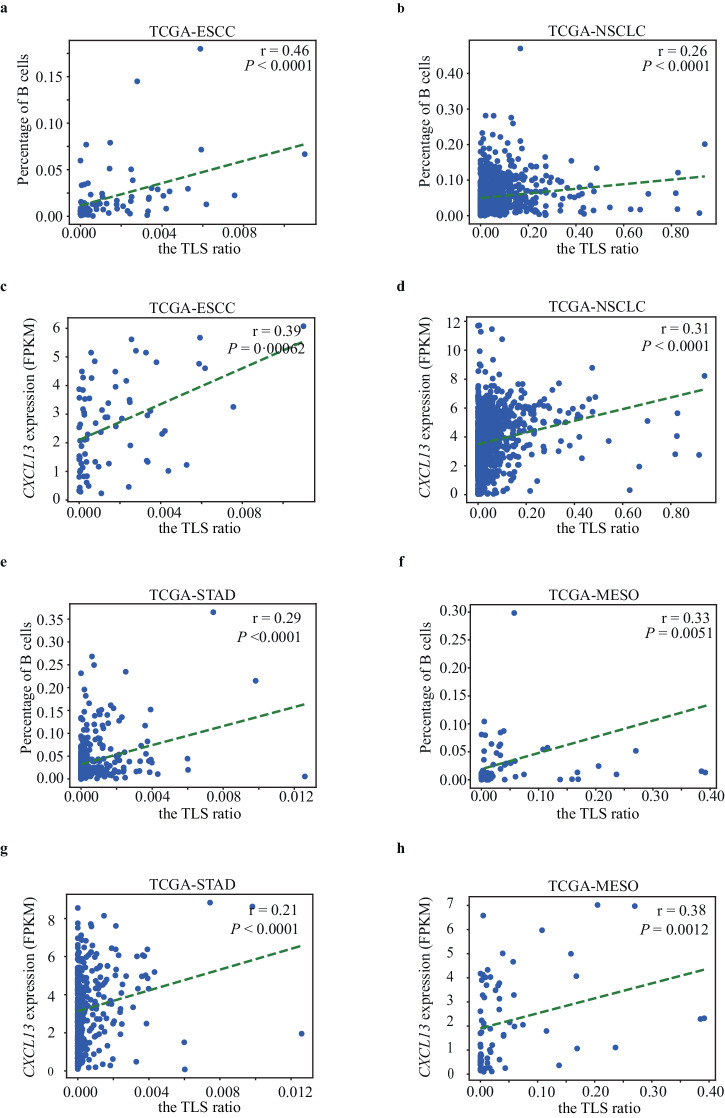
Correlation evaluations between the TLS ratios molecular signatures across tumor types from the TCGA. **a**, **b** Associations of the TLS ratios with the percentage of B lymphocytes in ESCC (**a**) and NSCLC (**b**) from the TCGA. **c**, **d** Associations of the TLS ratios with the expression of *CXCL13* in ESCC (**c**) and NSCLC (**d**) from the TCGA. **e**, **f** Associations of the TLS ratios with the percentage of B lymphocytes in STAD (**e**) and MESO (**f**) from the TCGA. **g**, **h** Associations of the TLS ratios with the expression of *CXCL13* in STAD (**g**) and MESO (**h**) from the TCGA. *P* values are calculated using a two-sided student’s *t* test. FPKM fragments per kilobase of transcript per Million mapped reads.

To evaluate the TLS ratios estimated by our approach, we first analyzed 74 ESCC and 936 NSCLC patients from the external TCGA. While mIHC data was unavailable for these patients, they had H&E WSIs along with RNA sequencing and DNA methylation data. We segmented TLSs (Supplementary Fig. 5) estimated TLS ratios from H&E WSIs, and compared them with molecular signatures reported to be correlated with TLSs (part III of Fig. 1). It has been shown that most tumor-infiltrating B lymphocytes aggregate inside TLSs^6^ and the number of B cells correlates with the number and area of TLSs^4^. By analyzing RNA sequencing data for gene expression levels and DNA methylation patterns, we were able to estimate the B cell percentages in the samples based on molecular signatures of B cell-specific genes. As expected, the estimated TLS ratios significantly correlated with the percentage of B lymphocytes in both ESCC (rho = 0.46, *P* value < 0.0001) (Fig. 2a) and NSCLC (rho = 0.26, *P* value < 0.0001) (Fig. 2b). TLS ratios also correlated with the expression of *CXCL13*, a chemokine associated with TLS formation^34^, in ESCC (rho = 0.39, *P* value = 0.0062) (Fig. 2c) and NSCLC (rho = 0.31, *P* value < 0.0001) (Fig. 2d).

Since TLS morphology is similar across cancers, we tested our approach in 14 additional TCGA tumor types (Supplementary Fig. 6). Similarly, estimated TLS ratios significantly correlated with B cell levels (Fig. 2e, f and Supplementary Fig. 7) and *CXCL13* expression in these cancers (Fig. 2g, h and Supplementary Fig. 8), suggesting broad applicability of our approach.

### Higher TLS ratios are associated with extended survival across various TCGA tumor types

**Fig. 3:**
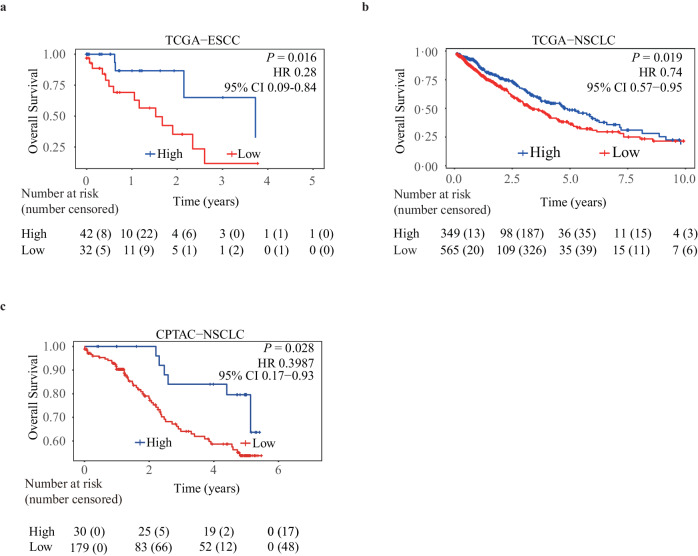
Overall survival outcomes stratified by the TLS ratios. TLS ratios estimated by our approach predict prognosis in ESCC (**a**), NSCLC (**b**) from the TCGA, and CPTAC-NSCLC (**c**) using univariate survival analysis. *P* values are calculated using a two-sided log-rank test.

TLSs have been identified as a potential prognostic indicator across multiple tumor types^1^. Thus, we explored the relationship between TLS ratios estimated from H&E WSIs and overall survival in various tumor types. Univariate survival analyses indicated that elevated TLS ratios correlated with prolonged overall survival in ESCC (hazard ratio [HR]: 0.28; 95% CI: 0.090–0.84; *P* value = 0.016) (Fig. 3a) and NSCLC (HR: 0.74; 95% CI: 0.57–0.95; *P* value = 0.019) (Fig. 3b) from TCGA. This was further validated in NSCLC cases from the Clinical Proteomic Tumor Analysis Consortium (CPTAC) (HR: 0.40; 95% CI: 0.17-0.93; *P* value = 0.028) (Fig. 3c). Subsequent multivariate analysis, adjusting for age, sex, and TNM staging (depth of invasion, lymph node metastasis and distant metastasis), confirmed that the positive association of TLS ratios with increased overall survival remains statistically significant for TCGA-ESCC, and marginally significant for TCGA-NSCLC and CPTAC-NSCLC (Supplementary Table 4).

In the other fourteen TCGA tumor types, ten of them also exhibited significant associations with univariate analyses (Supplementary Fig. 9, Supplementary Table 4). After adjusting for potential confounders, the associations remain significant for head and neck squamous cell carcinoma, prostate adenocarcinoma, colon and rectal cancer, and are marginally significant for liver hepatocellular carcinoma, skin cutaneous melanoma, pancreatic adenocarcinoma, testicular germ cell tumor (Supplementary Table 4). Moreover, the concordance index (C-index) values and *P* values obtained from the Cox regression models indicated that the inclusion of the TLS ratio with TNM staging significantly enhanced the models’ discriminative ability, outperforming the models that solely incorporated TNM staging, in 10 of 15 TCGA cancer types (Supplementary Table 5). Together, our findings underscore the potential of the TLS ratio as a prognostic biomarker in a range of solid tumors.

### Higher TLS ratios predicted a positive immunotherapy response across multiple cohorts

**Fig. 4:**
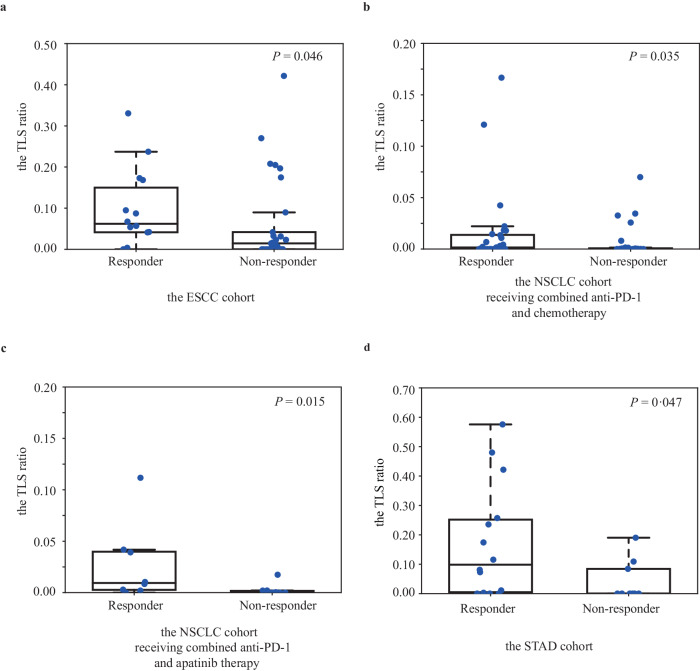
Assessment of the TLS ratios and immunotherapy outcomes in four independent cohorts. **a** The association between the TLS ratios and immunotherapy response in the ESCC cohort receiving anti-PD-1 monotherapy. **b** The association between the TLS ratios and immunotherapy response in the NSCLC cohort receiving combined anti-PD-1 and chemotherapy. **c** The association between the TLS ratios and immunotherapy response in the NSCLC cohort receiving combined anti-PD-1 and apatinib therapy. **d** The association between the TLS ratios and immunotherapy response in the STAD cohort receiving combining anti-PD-1 and concurrent chemoradiotherapy. *P* values are performed using a two-sided Wilcoxon rank-sum test. Box represents the median and the quartiles (lines). Whisker expresses 1.5 interquartile range of the lower or the upper quartile.

Finally, we assessed the TLS ratio as a biomarker for predicting clinical response to immunotherapy (part IV in Fig. 1). We estimated TLS ratios from H&E-stained biopsied tumor tissues before immunotherapy treatment. In an ESCC cohort (*n* = 43) receiving anti-PD-1 monotherapy in trial NCT02742935, TLS ratios were significantly higher in responders (33%, *n* = 14) versus non-responders (67%, *n* = 29) (*P* value = 0.046) (Fig. 4a). In two NSCLC cohorts given anti-PD-1 plus chemotherapy (*n* = 56) or anti-PD-1 plus apatinib (an antiangiogenic agent) (*n* = 18), TLS ratios were also significantly higher in responders compared to non-responders (*P* value = 0.035 and 0.015, respectively) (Fig. 4b, c). In a STAD cohort (n = 23) given anti-PD-1 and chemoradiotherapy, higher TLS ratio also associated with better immunotherapy response (*P* value = 0.047) (Fig. 4d). Overall, these data indicate the TLS ratio assessed by our deep learning approach on standard H&E histopathology images may provide useful prognostic and predictive insights across multiple tumor types.

## Discussion

Recently, several deep learning models have been developed towards automated segmentation of TLSs from H&E images in various tumor types, including lung cancer^35,36^ and gastrointestinal cancers^13^. Wang et al. extended its application by quantifying TLS density in lung adenocarcinoma tissues and explored its prognostic value^36^. Moreover, Rijthoven et al. introduced a multi-resolution strategy to segment and quantify TLSs, and applied these metrics as prognostic indicators in three distinct cancer types^37^, highlighting the versatility of computational models in different cancer contexts. Unlike these studies that depended solely on pathologists’ manual annotations of TLS without mIHC guidance, our study leveraged mIHC markers—DAPI, CD3, and CD20—to identify TLSs, thus reducing the influence of subjective human judgment. The robustness of the model was assessed through both internal and external validation sets. Additionally, we developed a pipeline to calculate the TLS ratio, enabling the automatic quantification of this metric. By employing our pipeline to thousands of patients from various external data sets, we demonstrated that estimated TLS ratios significantly correlated with established TLS-associated molecular signatures, including B cell abundance and *CXCL13* expression, suggesting the reliability of our approach to segment and quantify TLSs across multiple cancer types. More importantly, the derived TLS ratio holds promise as a robust pan-cancer biomarker that predicts prognosis and a positive immunotherapy response.

A major strength of our approach is the high-quality training dataset utilized, with TLS segmentation on H&E images verified through matched mIHC images and manually reviewed by experienced pathologists. This robust training process enabled the development of an automated, consistent model for precise TLS segmentation and quantification applicable to ubiquitous H&E slides, without reliance on specialized assays. Despite an imbalanced training dataset comprising sixty ESCC and five NSCLC tumor tissues, which might lead to better performance in ESCC, independent evaluation using the external validation set demonstrates that the IoU for each individual NSCLC case is still above 0.65 (Supplementary Fig. 4f), indicating satisfactory results. The strong correlation of estimated TLS ratios with established TLS biology across various TCGA tumor types provides confidence in its accuracy when applied to other cancer types. Moreover, this standardized segmentation methodology may also be generalizable for segmenting and quantifying TLSs in contexts beyond cancer, such as in autoimmune and infectious diseases. However, further benchmarking of the model’s TLS segmentation performance against mIHC in diverse diseases beyond ESCC and NSCLC would be valuable to formally validate its broader applicability.

TLSs are specialized lymphoid aggregates that often form in response to chronic inflammation^2^. They structurally and functionally resemble secondary lymphoid organs, supporting germinal center reactions that enable B cell activation and differentiation into plasma cells^2^. The presence of TLS has been linked to productive anti-tumor immunity in multiple cancers^38^. TLSs indicate an ongoing immune response and appear associated with better prognosis and immunotherapy outcomes across multiple cancers^2^. However, systematically evaluating TLSs currently requires multiplex imaging, which is resource-intensive and not widely available. Our study provides evidence that TLSs can be quantified through computational analysis of standard H&E histopathology images. Thus, it can be immediately applied to extract spatial and quantitative data on TLSs from abundant archival samples. Pairing these computationally derived TLS metrics with multi-omic data from the same samples provides an opportunity to uncover the molecular mechanisms governing TLS biology and its function in orchestrating anti-tumor immunity.

As immunotherapy expands, biomarkers to select patients and understand resistance mechanisms are urgently needed. Our data complements emerging evidence on the TLS ratio as an easily assessable pan-cancer biomarker predicting improved immunotherapy outcomes^2,4,17^. However, whether TLS ratios correlate with other established biomarkers, such as microsatellite instability^39^, PD-1/PD-L1 expression^40^, and tumor mutation burden^41^, warrants further study. In addition, separating mature from immature TLSs in our computational analysis could provide further biological insights and potentially better predict immunotherapy response. Mature TLSs with developed germinal centers likely promote stronger anti-tumor immunity compared to immature ones^11,42^. Dissecting these TLS subtypes may refine the utility of the TLS ratio as a predictive biomarker. Moreover, incorporating detailed clinical information about the cohorts analyzed and assessing relationships between computationally derived TLS metrics and other immunotherapy biomarkers could reveal if the TLS ratio gives orthogonal or synergistic value for predicting immunotherapy outcomes. This may enable improved response prediction compared to any single biomarker.

Another limitation of our study is that TLSs have complex 3D structures^43^, whereas we analyzed single 2D histological sections, which may not fully recapitulate the entire TLS immunologic composition, especially in small biopsied samples. While this methodological constraint is common and affects many types of histopathological analyses, 2D histology remains the standard in clinical settings due to its accessibility and feasibility. Studies have indicated that certain 2D image features can serve as surrogates for their 3D counterparts^44,45^, thus providing a feasible method for bridging the gap between practicality and accuracy. In fact, TLS areas calculated from 2D histological sections have been validated as biomarkers for prognosis and immunotherapy responses across various tumor types^13,46^. To mitigate this issue and better represent the 3D nature of TLSs, we propose the use of multiple non-consecutive sections from the same tumor to quantify and average the TLS ratios. Assessing TLSs across multiple standard histology images with our deep learning-based approach provides a practical way to better approximate real 3D TLS distribution while still relying on routine histopathology protocols. Further study with multi-slide analysis is warranted to validate improved performance over single-slide quantification. Additionally, the direct measurement of the number of TLSs from 2D histological sections may represent another metric that warrants further investigation.

Overall, we present a practical deep learning-based approach to extract clinically useful insights from H&E histopathology images. The TLS ratio provides a potential biomarker to stratify patients and illuminate cancer biology. Quantitative spatial analyses of the immune context using standard-of-care specimens could open avenues to improve immunotherapy.

## Methods

### Patients and data collection

Data used for the development of the TLS segmentation model was collected from surgically resected tumor tissues acquired from two distinct groups at the Zhongshan Hospital of Fudan University, Shanghai, China. Group one comprised sixty patients with ESCC, who underwent 1–4 cycles (28 days per cycle) of immunotherapy with combined anti-PD-1 blockade and chemotherapy. Group two consisted of five NSCLC patients who received 2 cycles of combined anti-PD-1 blockade and chemotherapy, with each cycle also lasting 28 days. The clinical characteristics of both groups are detailed in Supplementary Table 1.

In evaluating the TLS ratios, we employed the publicly available TCGA dataset. This dataset we used encompasses 6140 patients possessing H&E WSIs, concomitant RNA sequencing, and DNA methylation data. Detailed inclusion and exclusion criteria for each tumor types are described in Supplementary Fig. 10. Sixteen distinct tumor types were examined to evaluate correlations between estimated TLS ratios, molecular signatures, and prognosis. NSCLC cases from the Clinical Proteomic Tumor Analysis Consortium (CPTAC) used for the survival analysis encompass 960 H&E slides from 209 patients, which include both CPTAC-LSCC^47^ and CPTAC-LUAD^48^.

For evaluating the TLS ratio’s potential as an immunotherapy response predictor, we gathered data from four independent cohorts of ESCC, NSCLC, and STAD patients pre-therapy. The ESCC cohort (*n* = 43) was from a phase I clinical trial (NCT02742935)^49,50^. These patients, resistant or intolerant to prior chemotherapy, underwent 4 cycles (28 days per cycle) of treatment with the anti-PD-1 blockade (SHR-1210) at the Cancer Hospital of the Chinese Academy of Medical Science, Beijing, China. Treatment commenced at 60 mg and escalated to 200 mg and 400 mg, continuing until disease progression or the onset of intolerable side effects. Biopsied tumor tissues were procured as formalin-fixed paraffin-embedded (FFPE) samples before immunotherapy treatment. The clinical response for each patient was evaluated after treatment based on Response Evaluation Criteria in Solid Tumors (RECIST) v1.1^51^. Responders were defined as patients diagnosed with a complete response, and partial response; and non-responders were defined as patients diagnosed with stable disease, and progressive disease.

Two retrospective observational cohorts of NSCLC patients were gathered from the Cancer Hospital of the Chinese Academy of Medical Science, Beijing, China, from December 2021 to January 2023. The first cohort consisted of 56 patients who underwent combined anti-PD-1 blockade and chemotherapy treatments. The second cohort comprised 18 patients treated with a combination of anti-PD-1 blockade (camrelizumab) and an antiangiogenic agent (apatinib). All NSCLC patients underwent two cycles of immunotherapy treatment, each lasting 28 days, followed by surgical resection of tumor tissues 1-month post-treatment. Prior to initiating immunotherapy, FFPE tumor tissues were biopsied and subjected to H&E staining. The post-treatment clinical response for individuals in both cohorts was determined by expert pathologists who assessed the pathological response on surgically resected tumor specimens. Responders were characterized as patients manifesting over 90% tumor reduction^52^.

Data pertaining to the STAD cohort (*n* = 23) was retrospectively acquired from the Neo-PLANET phase II trial (NCT03631615)^53^, conducted at Zhongshan Hospital of Fudan University, Shanghai, China. Detailed inclusion and exclusion criteria are described in Supplementary Fig. 11. This investigation centered on immunotherapy, combining anti-PD-1 with concurrent chemoradiotherapy for patients with locally advanced adenocarcinoma of the stomach or gastroesophageal junction. The treatment protocol entailed the administration of anti-PD-1 blockade (capecitabine) at a dose of 850 mg/m^2^ twice daily, paired with concurrent radiotherapy spanning five weeks. This regimen was sandwiched by a 21-day cycle featuring oxaliplatin at 130 mg/m^2^ on day 1 and capecitabine at 1000 mg/m^2^ twice daily from days 1 to 14. Chemotherapy was concurrently administered over five cycles, each spanning 21 days, followed by surgical intervention after completing the total 15-week treatment period. Before treatment, tumor specimens were acquired through gastroscopy biopsy and subsequently stained with H&E. The post-treatment clinical responses of these patients were determined based on the expert pathologists’ assessment of the surgically resected tumor tissues. Responders in this cohort were identified as patients with a residual tumor cell count under 10%^53^. A detailed overview of these four cohorts is provided in Supplementary Table 3.

Every participant provided their informed written consent prior to their involvement in the study. All research procedures and protocols adhered to the principles set forth in the Declaration of Helsinki. Ethical approval for the study was granted by the Ethics Committees of the Cancer Hospital, Chinese Academy of Medical Science (Beijing, China), and the Zhongshan Hospital, Fudan University (Shanghai, China).

### Collecting of WSIs

Surgically resected tumor tissues from 60 ESCC patients and 5 NSCLC patients, processed as FFPE, were sectioned into 4 µm slides. These were subsequently stained for multiplex immunohistochemistry (mIHC) using rabbit anti-human monoclonal CD3 antibody (ab16669, Abcam) and mouse anti-human monoclonal CD20 antibody (14-0202-82, eBioscience). Post staining, the slides were treated with fluorescence mounting medium and underwent multispectral imaging at 20× magnification (0.5 μm/pixel) on the Vectra Polaris image system (Perkin Elmer). The channels designated for imaging included Opal 520 for CD3, Opal 690 for CD20, and DAPI for nuclei. These captured WSIs were subsequently visualized using Phenocart (Perkin Elmer).

For H&E staining, consecutive FFPE slides were deparaffinized in xylene and rehydrated through graded ethanol solutions. Slides were stained with Mayer’s hematoxylin for 5 minutes, followed by washing in running tap water for 5–10 minutes. Slides were differentiated in 1% acid alcohol briefly and blued in 0.2% ammonia water or Scott’s solution. Eosin counterstain was applied for 2 minutes. Following staining, slides were dehydrated through 95% and absolute alcohol, cleared in xylene, and mounted with resinous mounting medium.

To increase the robustness of the deep learning model across various H&E staining conditions, additional H&E staining was performed on consecutive slides from a random selection of nine ESCC patients. This involved variations in both the duration of hematoxylin staining and the frequency/duration of eosin incubation. The staining conditions were as follows:

- 2 slides were stained with Mayer’s hematoxylin for 8 minutes, followed by two eosin incubations of 2 minutes each.
- 2 slides stained with Mayer’s hematoxylin for 3 minutes, followed by a single eosin incubation of 1 minute.
- 2 slides stained with Mayer’s hematoxylin for 3 minutes, followed by two eosin incubations of 2 minutes each.
- 3 slides stained with Mayer’s hematoxylin for 8 minutes, followed by a single eosin incubation of 1 minute.

H&E-stained slides were digitized using a Perkin Elmer scanner at a magnification of 20×, resulting in a resolution of 0.5 μm/pixel. Additionally, 22 H&E slides were imaged at a magnification of 20× (0.5 μm/pixel) using two alternative scanner brands (KFBIO and Olympus) (Supplementary Table 2). Typically, it took approximately 5 minutes to digitize a H&E WSI. A total of 96 H&E WSIs, paired with 65 corresponding mIHC WSIs, were generated and utilized for the development of the TLS segmentation model.

### Processing of WSIs and TLS annotations in the internal data sets

TLS segmentation on the mIHC WSIs was conducted using the inForm image analysis software (Perkin Elmer). Briefly, all regions of interest (ROIs) spanning 930 μm × 697 μm, marked by aggregated lymphocytes based on CD3 and CD20 staining, were manually selected. The inForm software^54^ was used for cell segmentation, with the positivity thresholds for each marker set and cataloged for subsequent analyses. Selected ROIs underwent manual TLS segmentation based on CD3 and CD20 staining and used to establish a TLS segmentation algorithm to include at least 50 CD3^+^ or CD20^+^ lymphocytes. After the completion of the TLS segmentation algorithm, the remaining ROIs were batch-processed in the inForm, segregating them into TLS and non-TLS areas. The segmented ROIs were then mapped back into the WSIs to generate a comprehensive TLS segmentation of the mIHC WSIs.

Using the mIHC WSIs as ground truth, we manually generated TLS segmentation masks on the H&E WSIs. Post segmentation, two experienced pathologists (YQW with 12 years’ experience and DXJ with 10 years’ experience) performed the validation on the TLSs segmentation masks of the H&E WSIs using their mIHC counterparts. Using the OpenSlide Python package, H&E WSIs at 20× magnification and their corresponding TLS segmentation were cropped into 512 × 512-pixel tiles (256 μm × 256 μm) using a sliding window approach, retaining a 50% overlap. Only tiles with a TLS segmentation area exceeding 40% were curated. A total of 22,497 such tiles and their corresponding TLS segmentation were extracted from the 96 H&E WSIs (Supplementary Fig. 1). These tiles were then randomly divided into internal training, validation, and test sets in a ratio of 7:1:2, as detailed in Supplementary Table 2.

To segment TLS using the deep learning model, we kept the magnification of WSIs consistently at 20×. Each H&E WSI at 20× magnification was cropped into 512 × 512-pixel tiles (without overlap). For 40× magnification WSIs, 1024 × 1024-pixel tiles were cropped first, and then downscaled to the 512 × 512-pixel resolution.

### TLS annotations in the external validation set

From TCGA, we randomly selected five H&E-stained WSIs from ESCC and ten WSIs from NSCLC (including five lung adenocarcinoma and five lung squamous cell carcinoma). These WSIs were acquired either at 20× or 40× magnification and manually annotated by delineating the border of TLSs using the QuPath software^55^. The TLS segmentation annotations on these WSIs were validated by two experienced pathologists, YQW and DXJ, each with over a decade of professional experience in their field. Following this validation, H&E WSIs, together with their TLS segmentation annotations, were cropped into a total of 667 non-overlapping tiles to constitute the external validation set. The tile sizes were determined by their original magnification, with tiles from 20× magnification WSIs sized at 512 × 512 pixels, and those from 40× magnification WSIs at 1024 × 1024 pixels.

### Model development for TLS segmentation

TLSs are characterized by organized aggregations of T and B lymphocytes. Therefore, an optimal algorithm for TLS segmentation should capture the surrounding context of each cell to delineate a comprehensive TLS area. While numerous deep learning algorithms for medical image segmentation lean on UNet-like architectures, these often miss capturing pixel correlations across different channels due to their fusion of low-level textual and high-level semantic information. To address this, we adopted a previously described encoder-decoder model^31^, which incorporated two specially designed modules to capture contextual pixel correlations across various channels. Briefly, we chosen the EfficientNet-b0^56^ as the backbone of the TLS segmentation model. We used the AdamW optimizer^57^ to update the network parameters. We set the batch size to 64, the number of epochs to 100, and the learning rate and weight decay both to 1e-4, as described previously^31^. An early stopping operation was applied when the loss in the validation set did not decrease after 10 epochs. Both the internal training and validation sets were utilized exclusively for hyperparameter tuning. We adjusted the model parameters to achieve the best performance on the validation set. Once the optimal parameters were determined, the internal test set was then employed solely for the final evaluation of the model.

The performance of the TLS segmentation model was evaluated by the AUCs for the ROC curves in the internal training, validation, test sets, and external validation set (Supplementary Fig. 2). Briefly, we treated each image as a pixel-level binary classification task. Pixels identified as part of TLS were considered positive cases, while those not part of TLS were considered negative. We converted our model’s prediction probabilities into binary outcomes at various thresholds. This allowed us to calculate the True Positive Rate (TPR) and False Positive Rate (FPR), which facilitated the construction of the ROC curve and the computation of the AUC value.

In this study, pixels with a prediction probability for TLS segmentation above 0.5 were classified as part of the TLS area. The intersection over union was calculated by dividing the pixel count in the overlap between the predicted TLS area and the ground truth TLS area by the pixel count in the combined area of both. To compute the Dice Coefficient, we first doubled the pixel count in the intersection, then divided this by the total number of pixels present in both the predicted and the ground truth TLS area. The total TLS area used to compute the TLS ratio was the count of pixels predicted as belonging to the TLS area.

### Deep learning pipeline for TLS ratio calculation

The deep learning pipeline comprised three distinct branches, as illustrated in part II of Fig. 1. In addition to the branch to determine segmented TLS area, the pipeline comprised two branches designed to determine the lymphocyte count and the tissue area, respectively.

Segmentation and quantification of lymphocytes were executed using the publicly available deep learning model, HoVer-Net^33^. This model is adept at segmenting four distinct cell types from H&E WSIs, namely lymphocytes, macrophages, epithelial cells, neutrophils. We adopted the model pre-trained on the MoNuSAC2020 data sets^58^ to segment and enumerate lymphocyte counts. Tiles of varying resolutions—either $512\times 512$ pixels (0.5$\mu m/$ pixel, at $20\times$ magnification) or $1024\times 1024$ pixels (0.25$\mu m/$ pixel, at $40\times$ magnification) were resized to a dimension of $512\times 512$. A sliding window approach without overlap, measuring 256$\times$256 pixels, was then applied to segment cell instances. We noted a co-localization of segmented lymphocytes and TLSs (Supplementary Figs. 5 and 6), which emphasizes the model’s accuracy in detecting lymphocytes. By aggregating the results across all sliding windows, we enumerated the total lymphocyte count for each tile. In this study, tiles with a lymphocyte count exceeding 80 within the segmented TLSs were retained for the TLS ratio calculation.

Another branch to determine the tissue area employed the OTSU method^32^ from the OpenCV Python package to segment the tissue region from the non-tissue background. We applied various filters, including ‘filter_blue_pen’, ‘filter_green_pen’, and ‘filter_red_pen’ with default parameters from public codebase (https://github.com/deroneriksson/python-wsi-preprocessing), to eliminate annotations made using differently colored pens. The tissue area used to compute the TLS ratio was the count of pixels predicted as belonging to the tissue region. Tiles, wherein the segmented tissue area constitutes more than 10% of the entire tile area (equivalent to 26,214 pixels), were retained and processed further within the pipeline to compute the TLS ratio. For each WSI, the TLS ratio was derived by dividing the cumulative segmented TLS area by the total segmented tissue area. For subjects with more than one H&E WSIs, the TLS ratio was averaged among multiple WSIs.

### Estimate the percentage of B lymphocytes

For each patient in the TCGA, estimated percentage of B lymphocytes was determined by multiplying the overall leukocyte fraction with the estimated B cell proportion. Using CIBERSORT, we estimated proportions for twelve major immune cells from RNA-seq data. These cells included naive and memory B cells, naive, resting, and activated memory CD4 T cells, among others^59^. The estimated B cell proportion was a cumulative measure of naive, memory B cells, and plasma cells. The overall leukocyte fraction, derived from DNA methylation data, was obtained from the publicly released data^60^.

### Prognostic implications of TLS ratios in the TCGA and CPTAC

Upon estimating the TLS ratios for each patient in the TCGA, we utilized the surv_cutpoint function in the survminer R package to define the optimal cutoff, categorizing patients into high or low TLS ratio groups, in each cancer type^61^. This categorization was based on the highest standardized log-rank statistics. Both univariate and multivariate Cox regression analyses were conducted to evaluate the impact of TLS ratio categories on overall survival across various TCGA tumor types. Only patients who had complete data for adjusted variables, including sex (male versus female), age (above 60 years versus 60 years or below), and TNM staging, were included in the multivariate analysis (TCGA-SARC was excluded due to the lack of TNM staging). For both univariate and multivariate survival analyses in CPTAC-NSCLC, we used the optimal TLS ratio cutoff derived from the TCGA-NSCLC to stratify patients into high or low TLS ratio groups. 95% CIs were derived using the Wald test.

The C-indexes were determined in patients, who had complete data for TNM staging, across various TCGA tumor types. For these patients, C-indexes were calculated for three Cox regression models, each incorporating different sets of variables. The first model was based solely on the TLS ratio. The second model included the TNM staging, and the third model combined both TNM staging and the TLS ratio. A likelihood ratio test was performed to compare the nested Cox regression models, particularly between the second and the third models, to evaluate the incremental prognostic value of adding the TLS ratio to the conventional TNM staging in these tumor types.

### Statistical analyses

Hypothesis tests used to calculate *P* values were specified at corresponding figure legends and tables. The deep learning model’s performance was assessed using metrics such as the intersection over union, Dice coefficient, and CI. Survival curves were generated using the Kaplan–Meier method and compared using the log-rank test. To calculate the AUC in the TLS segmentation model, the 95% CI was calculated using 500 bootstrap replicates. Spearman’s correlation coefficients were employed for the TCGA to correlate TLS ratios with molecular signatures (B lymphocyte levels and *CXCL13* expression). A *P* value below 0.05 in a two-sided analysis was deemed significant. Analytical procedures were executed using Python (version 3.7.12), R (version 4.1.0), and the SciPy package (version 1.7.3)

### Hardware and software configuration

Our computational endeavors were predominantly facilitated by the PyTorch package (version 1.10.0). OpenSlide (version 1.2.0) was used to interpret WSIs. To convert images from various scanners into the ‘svs’ format compatible with OpenSlide, we employed the Pathomation software (version 2.0.0). Prognostic analyses were conducted using R (version 4.1.0). The SciPy python package (version 1.7.3) was employed for statistical evaluations. The R package ggsurvfit (version 0.3.0) was used to plot overall survival curves. For graphical illustrations, including dot and box plots, Matplotlib (version 3.5.3) was utilized.

The design, training, and assessment of our deep learning model were executed on a workstation with dual NVIDIA A100 GPUs, an AMD EPYC 7763 CPU (64 cores, 3.5 GHz), and 520 GB of random-access memory (RAM). On average, it took about 20 minutes to calculate the TLS ratio from an H&E WSI in the current settings.

### Reporting summary

Further information on research design is available in the Nature Research Reporting Summary linked to this article.

### Supplementary information

Supplementary Information

REPORTING SUMMARY

## Data Availability

The diagnostic whole-slide data and overall survival information from the TCGA and corresponding labels are available from NIH Genomic Data Commons (https://portal.gdc.cancer.gov/). The *CXCL13* expression data was obtained from UCSC XENA (https://xena.ucsc.edu/). The overall leukocyte fraction, derived from DNA methylation data, was obtained from a source (https://portal.gdc.cancer.gov/)^60^. The data sets of four cohorts receiving ICB therapy and TLS segmentation data sets, including H&E and mIHC WSIs, are available from the corresponding author upon reasonable request. The CPTAC-LSCC^47^ and CPTAC-LUAD^48^ were downloaded from The Cancer Imaging Archive (https://www.cancerimagingarchive.net/).

## Code availability

All the codes used in this work are available at: https://github.com/zonechen1994/AI_TLS_segmentation.

## References

[1] Schumacher TN, Thommen DS (2022). Tertiary lymphoid structures in cancer. Science.

[2] Sautes-Fridman C, Petitprez F, Calderaro J, Fridman WH (2019). Tertiary lymphoid structures in the era of cancer immunotherapy. Nat. Rev. Cancer.

[3] Helmink BA (2020). B cells and tertiary lymphoid structures promote immunotherapy response. Nature.

[4] Fridman WH (2022). B cells and tertiary lymphoid structures as determinants of tumour immune contexture and clinical outcome. Nat. Rev. Clin. Oncol..

[5] Rodriguez AB, Engelhard VH (2020). Insights into tumor-associated tertiary lymphoid structures: novel targets for antitumor immunity and cancer immunotherapy. Cancer Immunol. Res..

[6] Petitprez F (2020). B cells are associated with survival and immunotherapy response in sarcoma. Nature.

[7] Cabrita R (2020). Tertiary lymphoid structures improve immunotherapy and survival in melanoma. Nature.

[8] Italiano A (2022). Pembrolizumab in soft-tissue sarcomas with tertiary lymphoid structures: a phase 2 PEMBROSARC trial cohort. Nat. Med..

[9] Yu A (2023). The prognostic value of the tertiary lymphoid structure in gastrointestinal cancers. Front. Immunol..

[10] Sun H (2023). Prognostic value of tertiary lymphoid structures (TLS) in digestive system cancers: a systematic review and meta-analysis. BMC Cancer.

[11] Vanhersecke L (2021). Mature tertiary lymphoid structures predict immune checkpoint inhibitor efficacy in solid tumors independently of PD-L1 expression. Nat. Cancer.

[12] Goff PH (2022). Neoadjuvant therapy induces a potent immune response to sarcoma, dominated by myeloid and B cells. Clin. Cancer Res..

[13] Li Z (2023). Development and validation of a machine learning model for detection and classification of tertiary lymphoid structures in gastrointestinal cancers. JAMA Netw. Open.

[14] Ling Y (2022). The prognostic value and molecular properties of tertiary lymphoid structures in oesophageal squamous cell carcinoma. Clin. Transl. Med..

[15] Bulten W (2020). Automated deep-learning system for Gleason grading of prostate cancer using biopsies: a diagnostic study. Lancet Oncol..

[16] Wang Y (2022). Improved breast cancer histological grading using deep learning. Ann. Oncol..

[17] Crombe A, Roulleau-Dugage M, Italiano A (2022). The diagnosis, classification, and treatment of sarcoma in this era of artificial intelligence and immunotherapy. Cancer Commun. (Lond.).

[18] Bera K, Schalper KA, Rimm DL, Velcheti V, Madabhushi A (2019). Artificial intelligence in digital pathology - new tools for diagnosis and precision oncology. Nat. Rev. Clin. Oncol..

[19] Lee, Y. et al. Derivation of prognostic contextual histopathological features from whole-slide images of tumours via graph deep learning. *Nat. Biomed. Eng.*10.1038/s41551-022-00923-0 (2022). 10.1038/s41551-022-00923-0 35982331

[20] Skrede OJ (2020). Deep learning for prediction of colorectal cancer outcome: a discovery and validation study. Lancet.

[21] Wang S (2020). Computational staining of pathology images to study the tumor microenvironment in lung cancer. Cancer Res..

[22] Rakaee M (2023). Association of machine learning-based assessment of tumor-infiltrating lymphocytes on standard histologic images with outcomes of immunotherapy in patients with NSCLC. JAMA Oncol..

[23] Johannet P (2021). Using machine learning algorithms to predict immunotherapy response in patients with advanced melanoma. Clin. Cancer Res..

[24] Jiang Y (2023). Biology-guided deep learning predicts prognosis and cancer immunotherapy response. Nat. Commun..

[25] Shamai G (2022). Deep learning-based image analysis predicts PD-L1 status from H&E-stained histopathology images in breast cancer. Nat. Commun..

[26] Shamai G (2019). Artificial intelligence algorithms to assess hormonal status from tissue microarrays in patients with breast cancer. JAMA Netw. Open.

[27] Coudray N (2018). Classification and mutation prediction from non-small cell lung cancer histopathology images using deep learning. Nat. Med..

[28] Liao H (2020). Deep learning-based classification and mutation prediction from histopathological images of hepatocellular carcinoma. Clin. Transl. Med..

[29] Li D (2020). A deep learning diagnostic platform for diffuse large B-cell lymphoma with high accuracy across multiple hospitals. Nat. Commun..

[30] Xu Y (2017). Large scale tissue histopathology image classification, segmentation, and visualization via deep convolutional activation features. BMC Bioinformatics.

[31] Chen, Z., Wang, K. & Liu, Y. Efficient polyp segmentation via integrity learning. *ICASSP 2024 - 2024 IEEE International Conference on Acoustics, Speech and Signal Processing (ICASSP)* 1826–1830 (2024).

[32] Otsu N (1979). A threshold selection method from gray-level histograms. IEEE Trans. Syst. Man Cybern..

[33] Graham S (2019). Hover-Net: simultaneous segmentation and classification of nuclei in multi-tissue histology images. Med Image Anal..

[34] Rouanne M, Arpaia N, Marabelle A (2021). CXCL13 shapes tertiary lymphoid structures and promotes response to immunotherapy in bladder cancer. Eur. J. Cancer.

[35] Barmpoutis P (2021). Tertiary lymphoid structures (TLS) identification and density assessment on H&E-stained digital slides of lung cancer. PLoS One.

[36] Wang Y (2023). Computerized tertiary lymphoid structures density on H&E-images is a prognostic biomarker in resectable lung adenocarcinoma. iScience.

[37] van Rijthoven M (2024). Multi-resolution deep learning characterizes tertiary lymphoid structures and their prognostic relevance in solid tumors. Commun. Med. (Lond.).

[38] Meylan M (2022). Tertiary lymphoid structures generate and propagate anti-tumor antibody-producing plasma cells in renal cell cancer. Immunity.

[39] Florou, V. et al. Real-world pan-cancer landscape of frameshift mutations and their role in predicting responses to immune checkpoint inhibitors in cancers with low tumor mutational burden. *J. Immunother. Cancer* **11**, e007440 (2023). 10.1136/jitc-2023-007440 PMC10432623 37586768

[40] Lu S (2019). Comparison of biomarker modalities for predicting response to PD-1/PD-L1 checkpoint blockade: a systematic review and meta-analysis. JAMA Oncol..

[41] Chan TA (2019). Development of tumor mutation burden as an immunotherapy biomarker: utility for the oncology clinic. Ann. Oncol..

[42] Brunet M (2022). Mature tertiary lymphoid structure is a specific biomarker of cancer immunotherapy and does not predict outcome to chemotherapy in non-small-cell lung cancer. Ann. Oncol..

[43] Mai, H. et al. Whole-body cellular mapping in mouse using standard IgG antibodies. *Nat. Biotechnol.*10.1038/s41587-023-01846-0 (2023). 10.1038/s41587-023-01846-0 PMC11021200 37430076

[44] Lee KH (2014). Correlation between the size of the solid component on thin-section CT and the invasive component on pathology in small lung adenocarcinomas manifesting as ground-glass nodules. J. Thorac. Oncol..

[45] Yang WT, Tse GM, Lam PK, Metreweli C, Chang J (2002). Correlation between color power Doppler sonographic measurement of breast tumor vasculature and immunohistochemical analysis of microvessel density for the quantitation of angiogenesis. J. Ultrasound Med..

[46] van Dijk N (2020). Preoperative ipilimumab plus nivolumab in locoregionally advanced urothelial cancer: the NABUCCO trial. Nat. Med..

[47] Consortium, N.C.I.C.P.T.A. The clinical proteomic tumor analysis consortium lung squamous cell carcinoma collection (CPTAC-LSCC), (The Cancer Imaging Archive, 2018).

[48] Consortium, N.C.I.C.P.T.A. The clinical proteomic tumor analysis consortium lung adenocarcinoma collection (CPTAC-LUAD), (2018).

[49] Liu Z (2023). Integrated multi-omics profiling yields a clinically relevant molecular classification for esophageal squamous cell carcinoma. Cancer Cell.

[50] Huang J (2018). Safety, activity, and biomarkers of SHR-1210, an anti-PD-1 antibody, for patients with advanced esophageal carcinoma. Clin. Cancer Res..

[51] Eisenhauer EA (2009). New response evaluation criteria in solid tumours: revised RECIST guideline (version 1.1). Eur. J. Cancer.

[52] Vos JL (2021). Neoadjuvant immunotherapy with nivolumab and ipilimumab induces major pathological responses in patients with head and neck squamous cell carcinoma. Nat. Commun..

[53] Tang Z (2022). The Neo-PLANET phase II trial of neoadjuvant camrelizumab plus concurrent chemoradiotherapy in locally advanced adenocarcinoma of stomach or gastroesophageal junction. Nat. Commun..

[54] Kramer AS (2018). InForm software: a semi-automated research tool to identify presumptive human hepatic progenitor cells, and other histological features of pathological significance. Sci. Rep..

[55] Bankhead P (2017). QuPath: open source software for digital pathology image analysis. Sci. Rep..

[56] Tan, M. & Le, Q. V. EfficientNet: rethinking model scaling for convolutional neural networks. *In:* Proceedings of the 36th international conference on machine learning, https://arxiv.org/abs/1905.11946 (2019).

[57] Loshchilov, I. & Hutter, F. Decoupled Weight Decay Regularization. *In:* 7th international conference on learning representations, https://arxiv.org/abs/1711.05101 (2019).

[58] Verma R (2021). MoNuSAC2020: a multi-organ nuclei segmentation and classification challenge. IEEE Trans. Med. Imaging.

[59] Newman AM (2015). Robust enumeration of cell subsets from tissue expression profiles. Nat. Methods.

[60] Thorsson V (2019). The immune landscape of cancer. Immunity.

[61] Chen D (2022). Prognostic and predictive value of a pathomics signature in gastric cancer. Nat. Commun..

