# Supplementary material for: Deep learning on tertiary lymphoid structures in hematoxylin-eosin predicts cancer prognosis and immunotherapy response
Source: NPJ Precis Oncol. 2024 Mar 22;8:73. doi: 10.1038/s41698-024-00579-w (PMC10959936; doi:10.1038/s41698-024-00579-w)
Supplement: Supplementary file 1 — Supplementary Information [file 41698_2024_579_MOESM1_ESM.pdf]

Supplementary Figure Legends

Supplementary Figure 1 Examples of TLS segmentation masks on the H&E tiles based on their mIHC counterparts. The left column: mIHC staining of CD20, CD3, together with DAPI. The middle column: H&E staining of consecutive slides. The right column: TLS segmentation masks on the H&E tiles (the middle column) based on their mIHC counterparts (the left column). Five examples were illustrated (**a - e**). Scale bar: 100 μm.

Supplementary Figure 2 The AUC of the receiver operator characteristic of the TLS segmentation model in our dataset. (**a**) The performance in the training set. (**b**) The performance in the validation set. (**c**) The performance in the test set. (**d**) The performance in the external validation set. The 95% CI of the AUC was calculated using the bootstrap method.

Supplementary Figure 3 Examples of predicted TLS segmentations on the test set. The left column: mIHC staining of CD20, CD3, together with DAPI. The middle column: H&E staining of consecutive slides. The right column: predicted TLS segmentations on the H&E tiles (the middle column) generated by the TLS segmentation model. Five examples were illustrated (**a - e**). Scale bar: 100 μm.

Supplementary Figure 4 Evaluation of the model's prediction accuracy at both the tile and slide levels across internal and external data sets. (**a - c**) Correlations between the predicted and observed TLS area for each tile in the internal training (**a**), validation (**b**), and test (**c**) sets. (**d**) An intersection over union (IoU) for each slide in the internal data set. (**e**) The correlation between the predicted and observed TLS area for each tile in the external validation set. (**f**) An intersection over union (IoU) for each slide in the external validation set. *P* values were calculated using a two-sided student's t test.

Supplementary Figure 5 Examples of predicted lymphocyte TLS segmentations in ESCC and NSCLC patients from the TCGA. The left column: H&E staining results. The middle column: predicted lymphocyte segmentations on the H&E tiles (the left column) generated by the HoVer-Net. The right column: predicted TLS segmentations on the H&E tiles (the left column) generated by the TLS segmentation model. Examples of four ESCC (**a - d**), two LUAD (**e, f**) and two LUSC (**g, h**) were illustrated. ESCC = esophageal squamous cell carcinoma, NSCLC = non-small cell lung cancer, LUAD = lung adenocarcinoma, LUSC = lung squamous cell carcinoma. Scale bar: 100 μm.

Supplementary Figure 6 Examples of predicted lymphocyte and TLS segmentations across multiple TCGA tumor types. The left column: H&E staining results. The middle column: predicted lymphocyte segmentations on the H&E tiles (the left column) generated by the HoVer-Net. The right column: predicted TLS segmentations on the H&E tiles (the left column) generated by the TLS segmentation model. (**a**) STAD, (**b**) MESO, (**c**) BRCA, (**d**) HNSC, (**e**) PRAD, (**f**) BLCA, (**g**) SARC, (**h**) LIHC, (**i**) COAD, (**j**) SKCM, (**k**) PAAD, (**l**) CHOL, (**m**) THCA and (**n**) TGCT from the TCGA. STAD = stomach adenocarcinoma, MESO = mesothelioma, BRCA = breast invasive carcinoma, HNSC = head and neck squamous cell carcinoma, PRAD = prostate adenocarcinoma, BLCA = bladder urothelial carcinoma, SARC = sarcoma, LIHC = liver hepatocellular carcinoma, CRC = colon and rectal cancer, SKCM = skin cutaneous melanoma, PAAD = pancreatic adenocarcinoma, CHOL = cholangiocarcinoma, THCA = thyroid carcinoma, TGCT = testicular germ cell tumor. Scale bar: 100 μm.

Supplementary Figure 7 Associations of the TLS ratios with the percentage of B lymphocytes. (**a**) BRCA, (**b**) HNSC, (**c**) PRAD, (**d**) BLCA, (**e**) SARC, (**f**) LIHC, (**g**) CRC, (**h**) SKCM, (**i**) PAAD, (**j**) CHOL, (**k**) THCA and (**l**) TGCT from the TCGA.

Supplementary Figure 8: Associations of the TLS ratios with the expression of *CXCL13*. (**a**) BRCA, (**b**) HNSC, (**c**) PRAD, (**d**) BLCA, (**e**) SARC, (**f**) LIHC, (**g**) CRC, (**h**) SKCM, (**i**) PAAD, (**j**) CHOL, (**k**) THCA and (**l**) TGCT from the TCGA. FPKM: Fragments Per Kilobase of transcript per Million mapped reads.

Supplementary Figure 9 Overall survival outcomes stratified by the TLS ratios. TLS ratios estimated by our approach predict prognosis for patients in (**a**) BRCA, (**b**) STAD, (**c**)MESO, (**d**) BLCA, (**e**) SARC, (**f**) LIHC, (**g**) CRC, (**h**) SKCM, (**i**) PAAD, (**j**) CHOL, (**k**) THCA, (**l**) TGCT, (**m**) HNSC, and (**n**) PRAD from the TCGA using a univariate survival analysis. *P* values were calculated using a two-sided log-rank test.

Supplementary Figure 10 Flow chart of patient inclusion and exclusion across multiple TCGA tumor types. (**a**) ESCC, (**b**) NSCLC, (**c**) STAD, (**d**) MESO, (**e**) BRCA, (**f**) HNSC, (**g**) PRAD, (**h**) BLCA, (**i**) SARC, (**j**) LIHC, (**k**) CRC, (**l**) SKCM, (**m**) PAAD, (**n**) CHOL, (**o**) THCA, and (**p**) TGCT.

Supplementary Figure 11 Flow chart of patient inclusion and exclusion in the STAD cohort.

Supplementary Figure 1

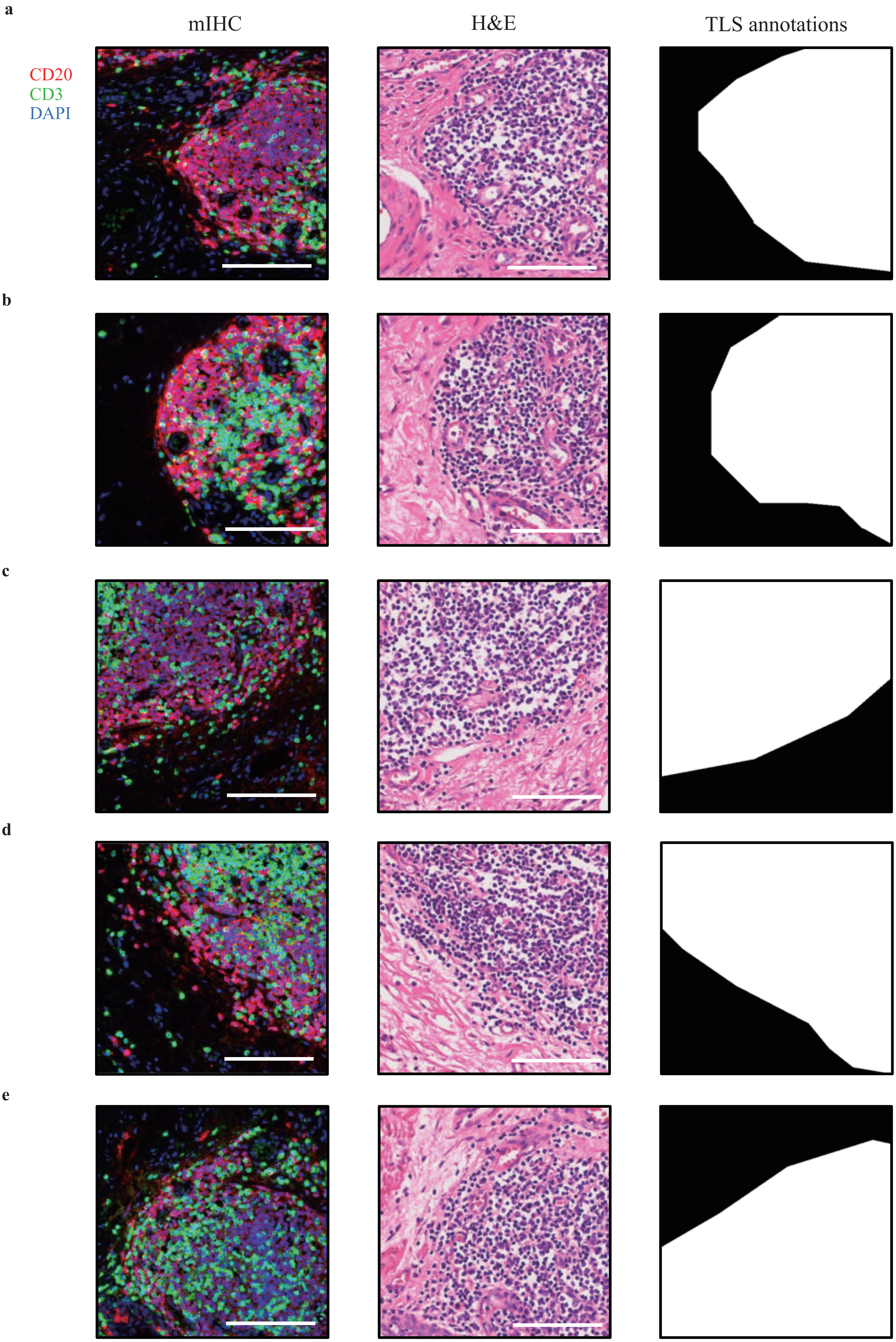

Supplementary Figure 2

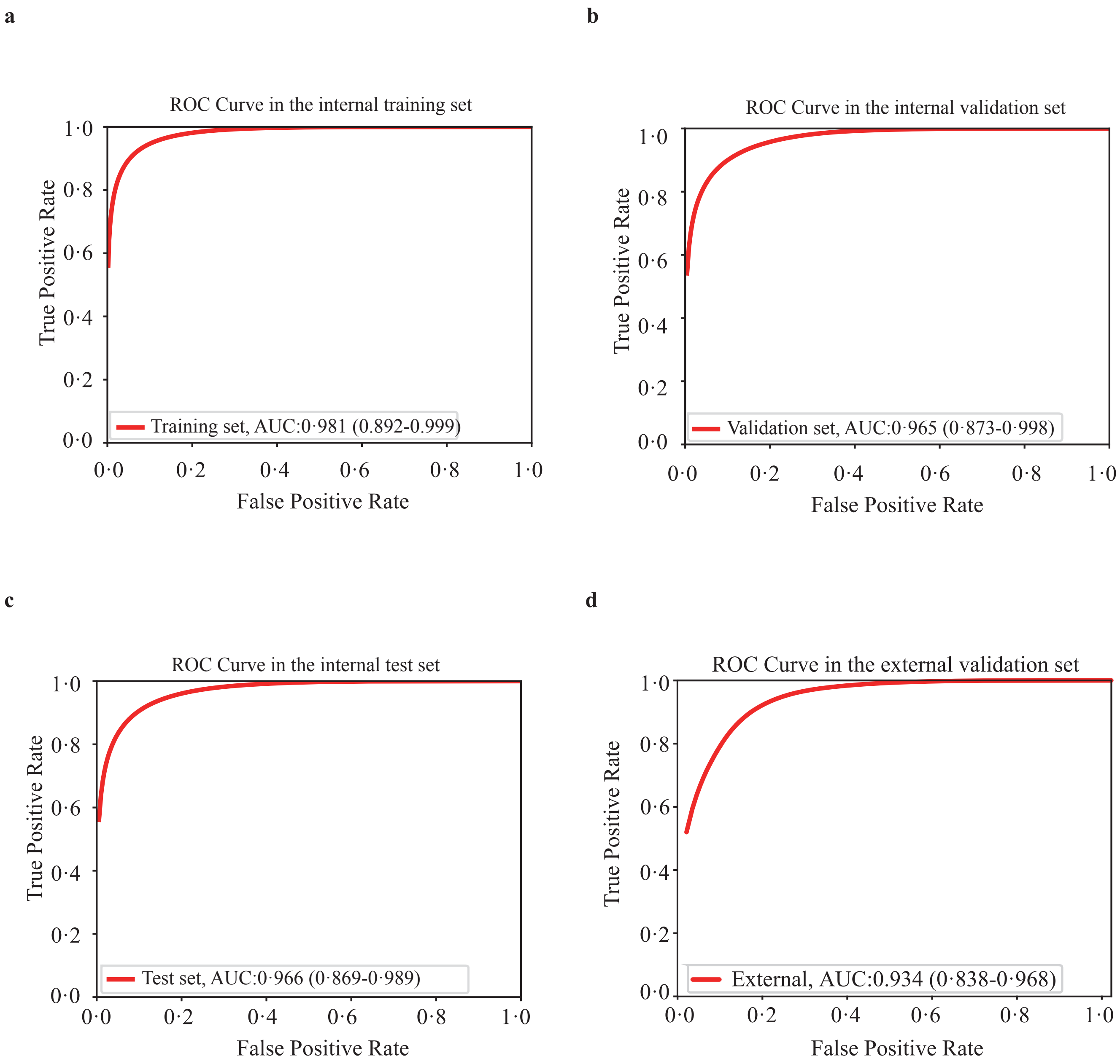

Supplementary Figure 3

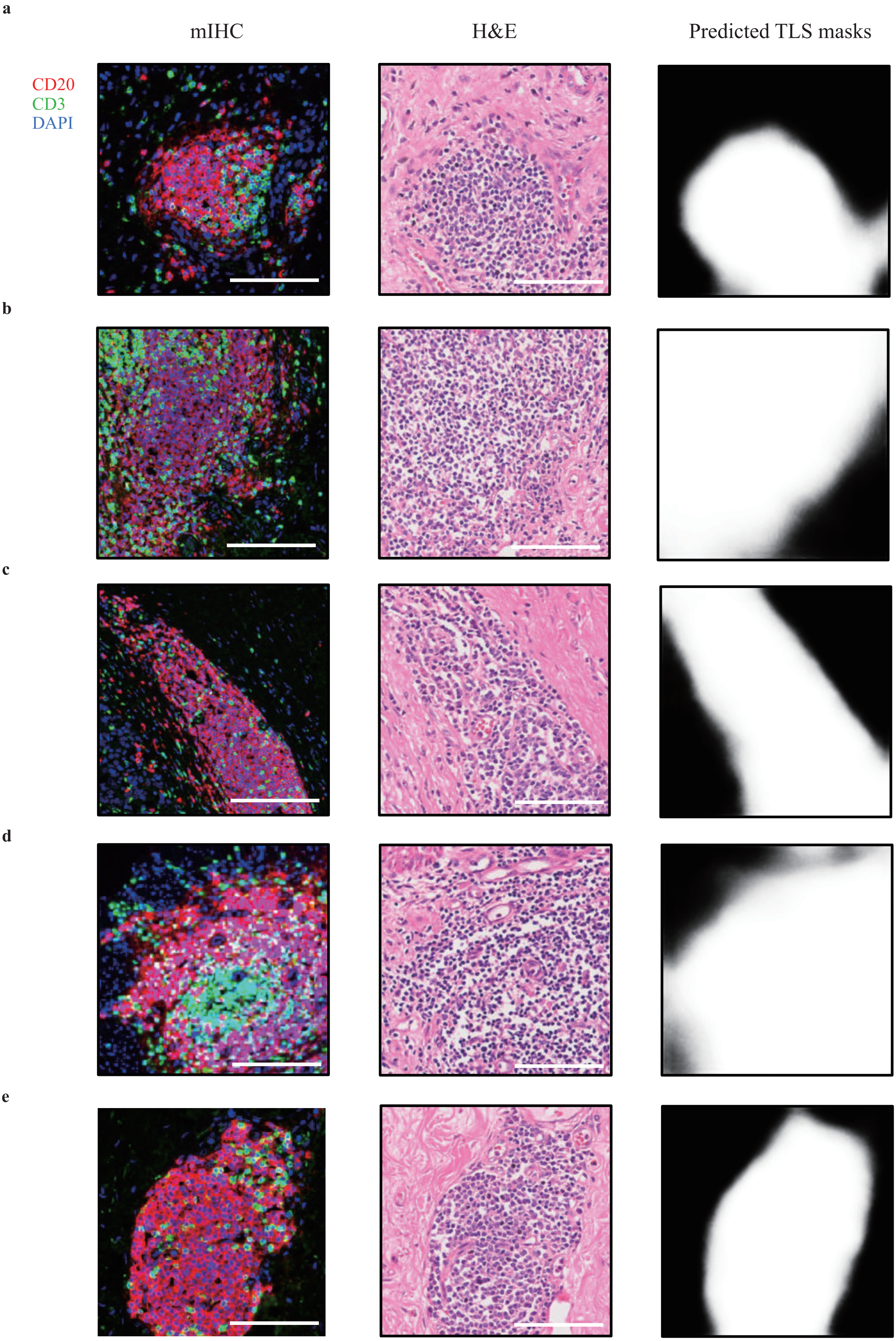

Supplementary Figure 4

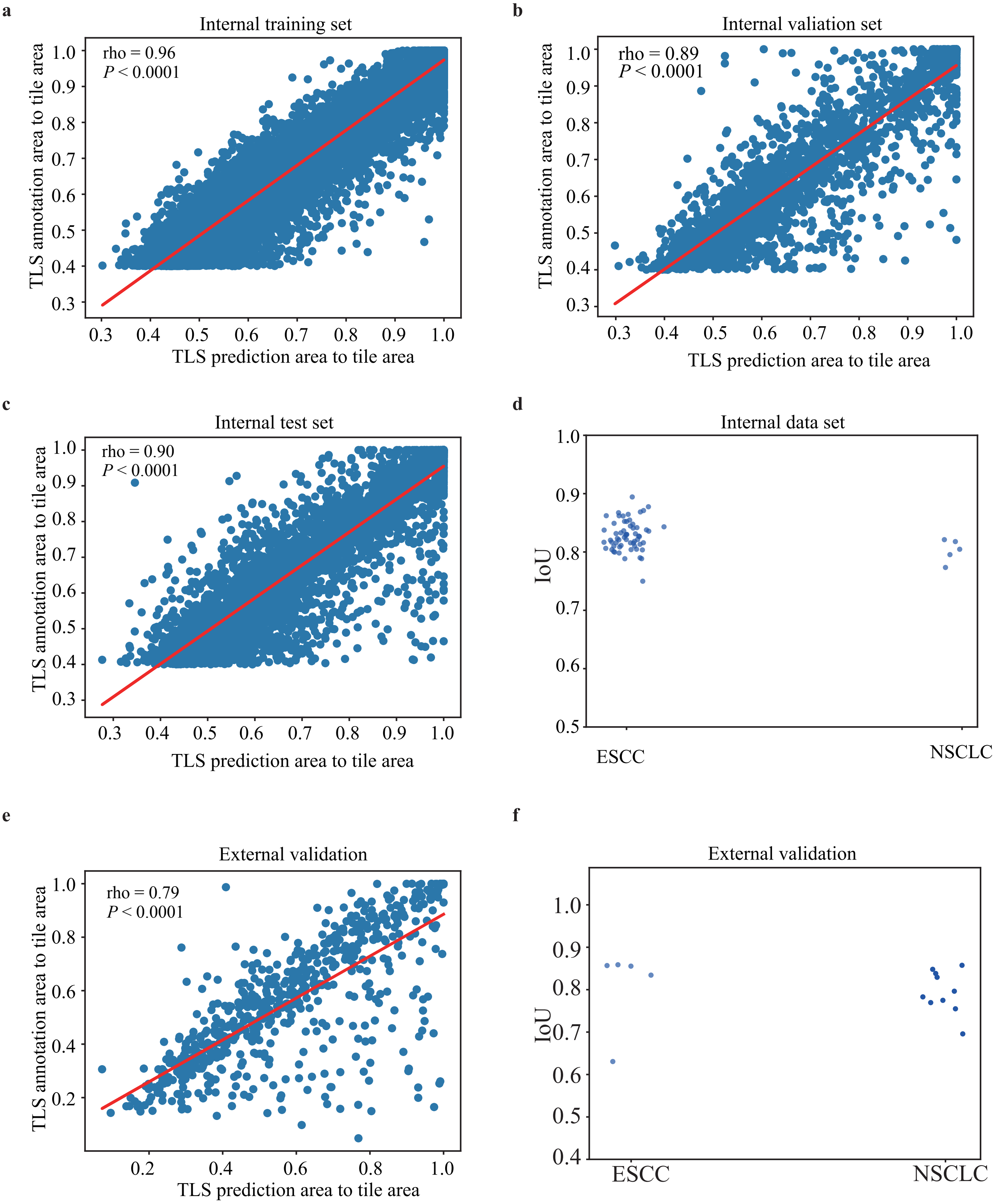

Supplementary Figure 5

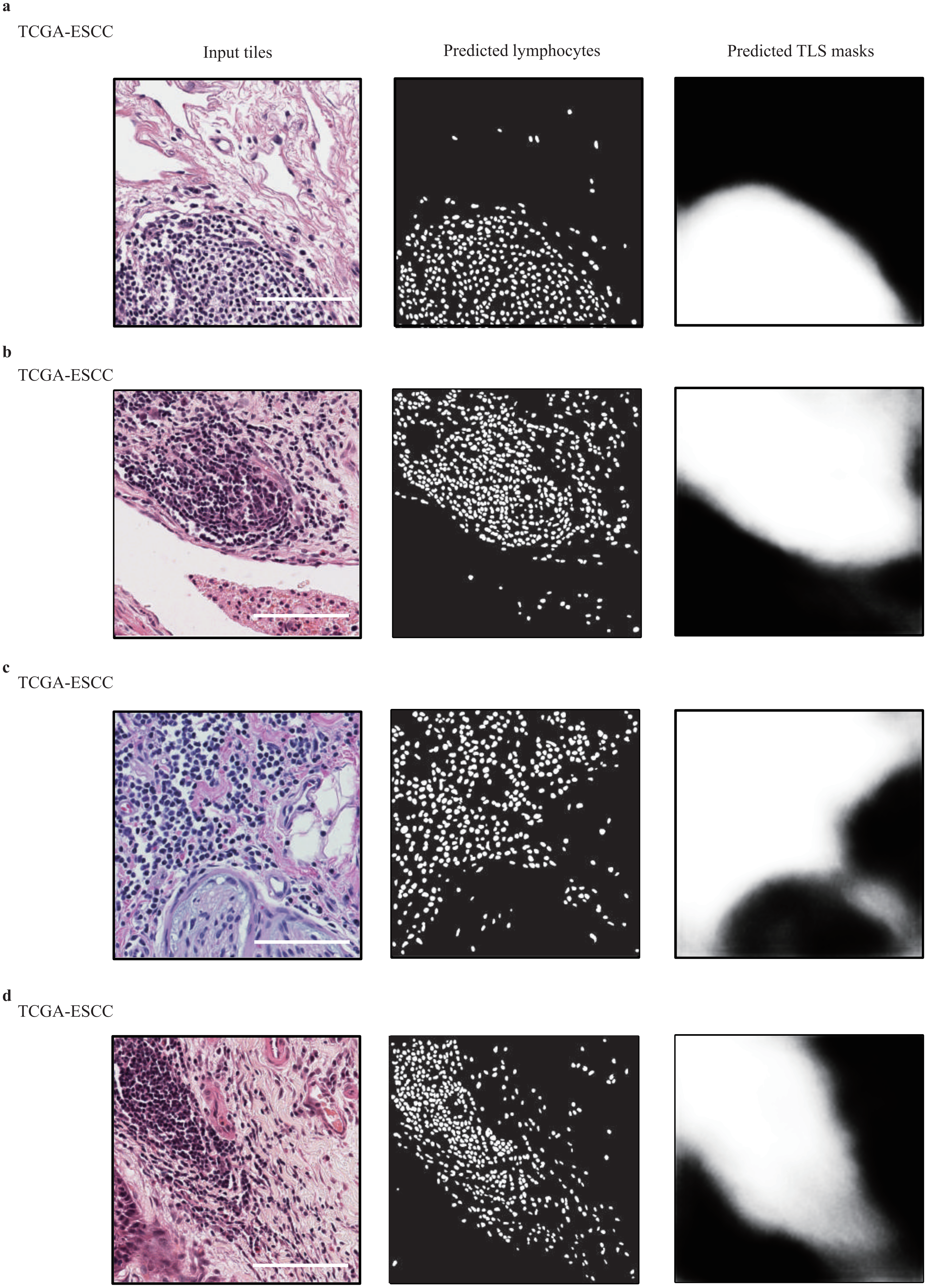

Supplementary Figure 5 (continued)

e

TCGA-LUAD

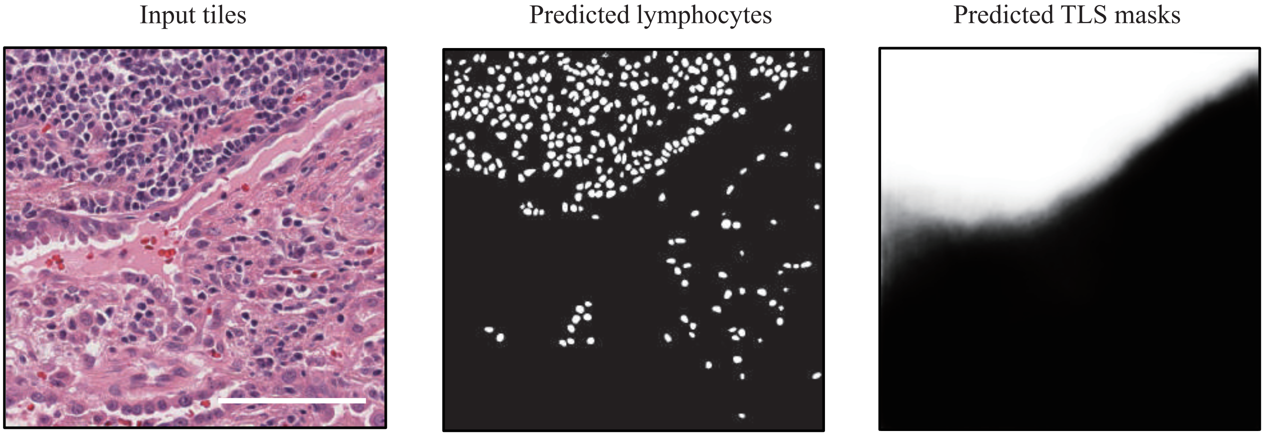

f

TCGA-LUAD

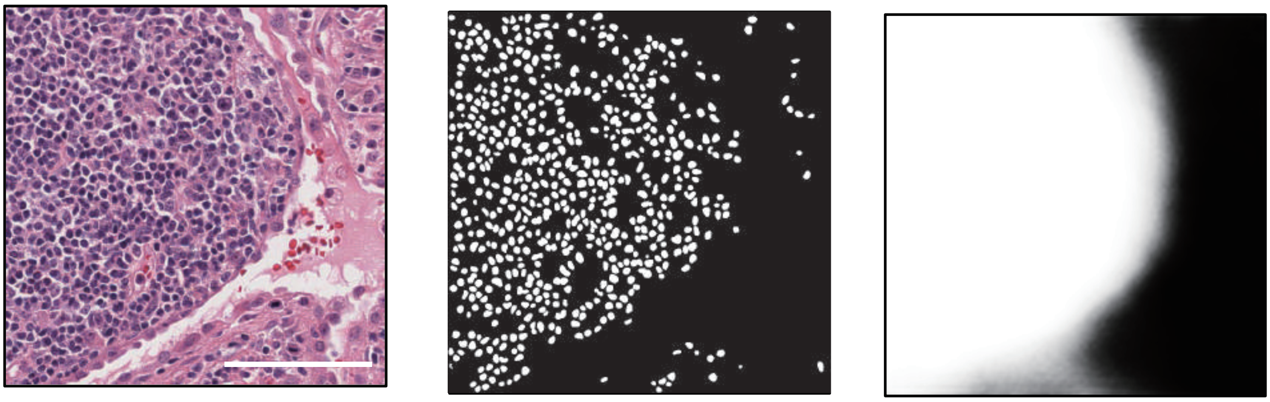

g

TCGA-LUSC

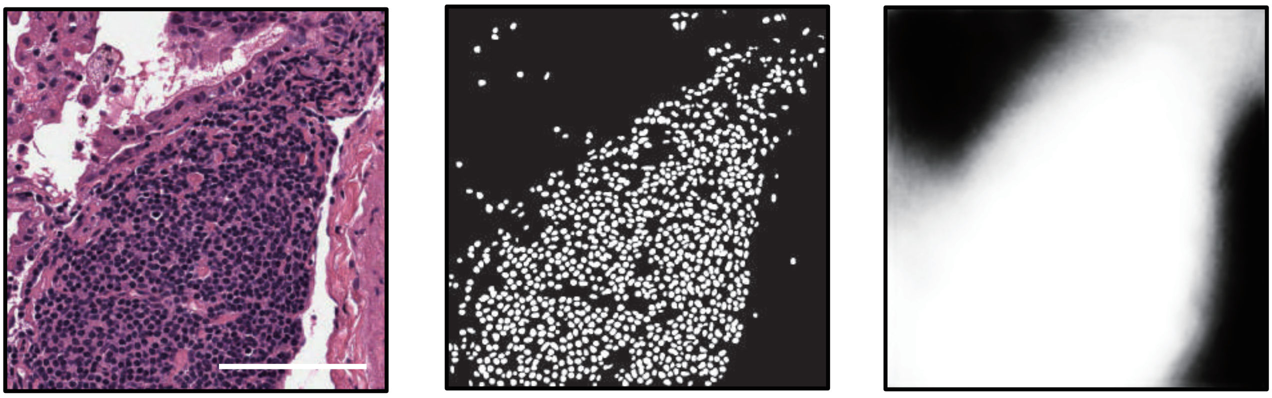

h

TCGA-LUSC

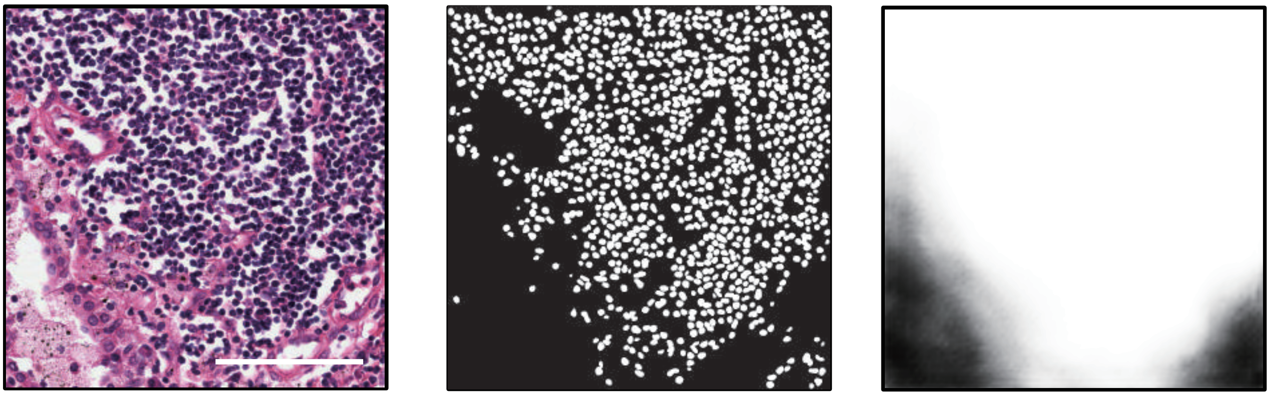

Supplementary Figure 6

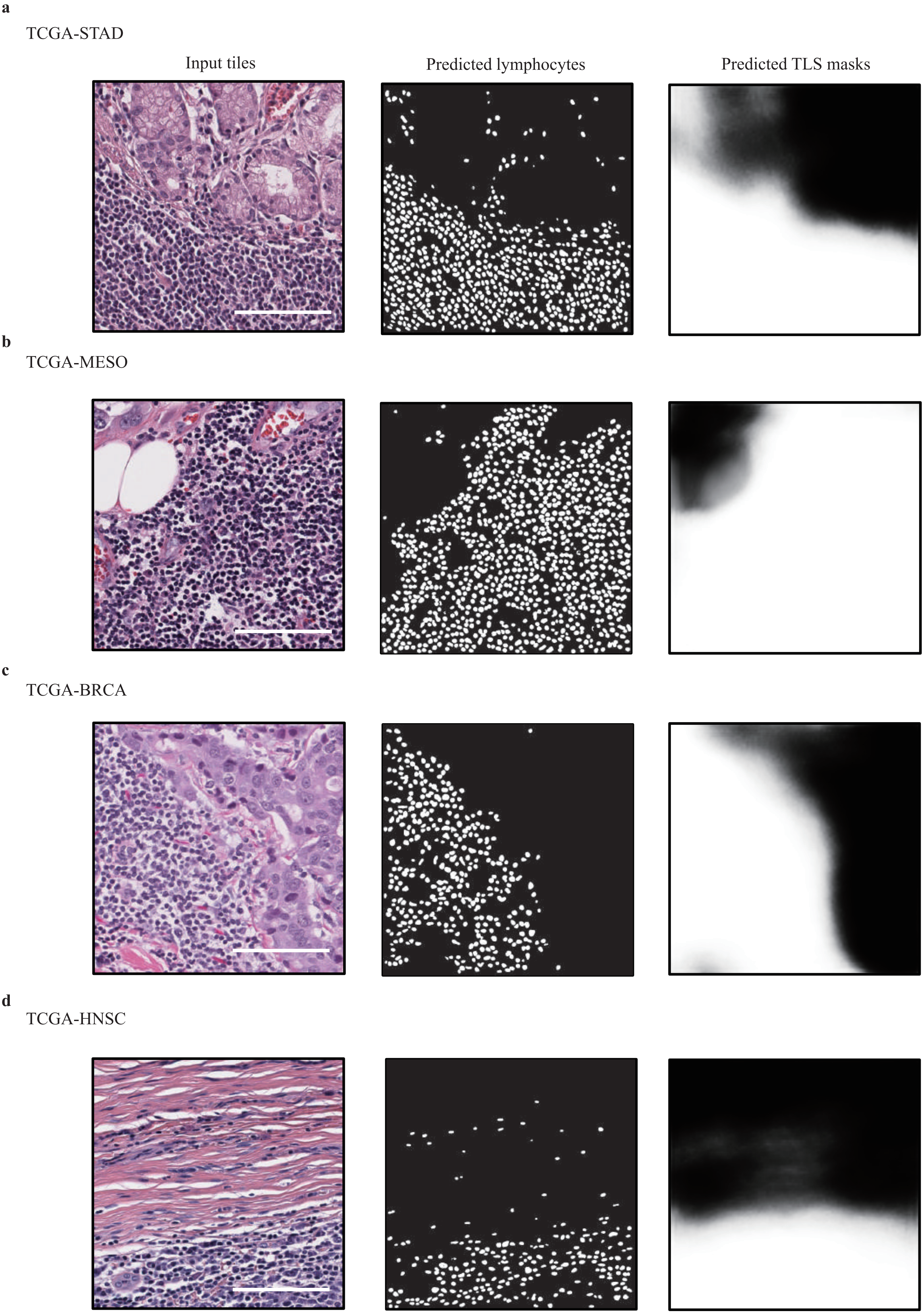

Supplementary Figure 6 (continued)

e

TCGA-PRAD

Input tiles | Predicted lymphocytes | Predicted TLS masks

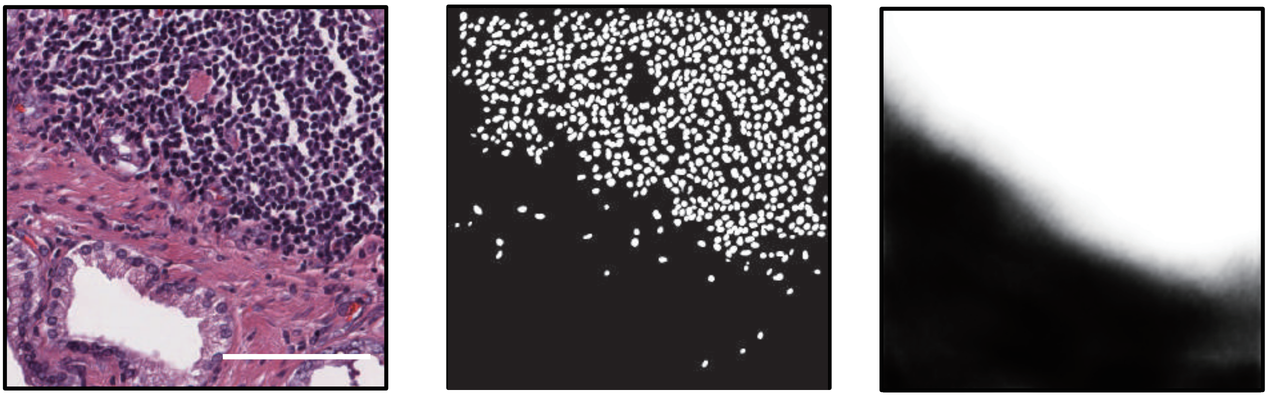

f

TCGA-BLCA

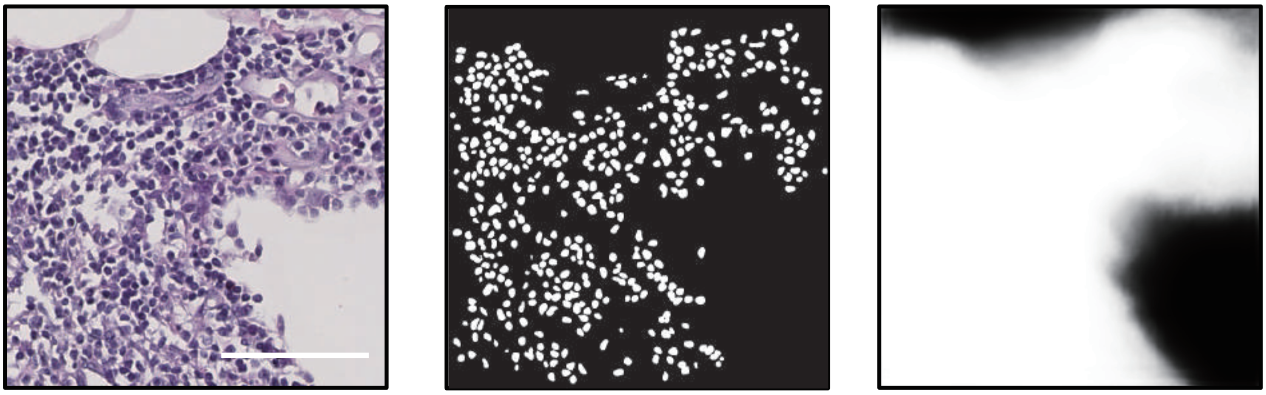

g

TCGA-SARC

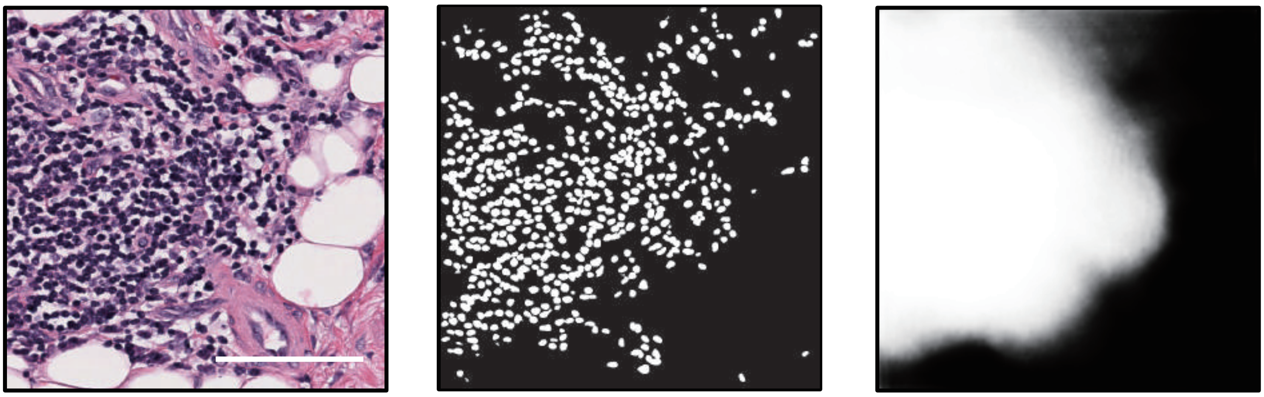

h

TCGA-LIHC

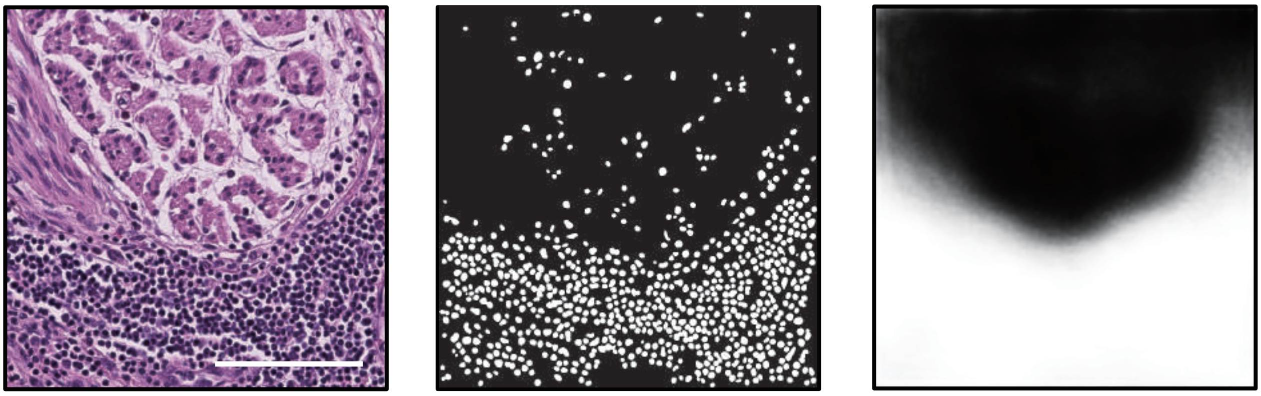

Supplementary Figure 6 (continued)

i

TCGA-COAD

Input tiles | Predicted lymphocytes | Predicted TLS masks

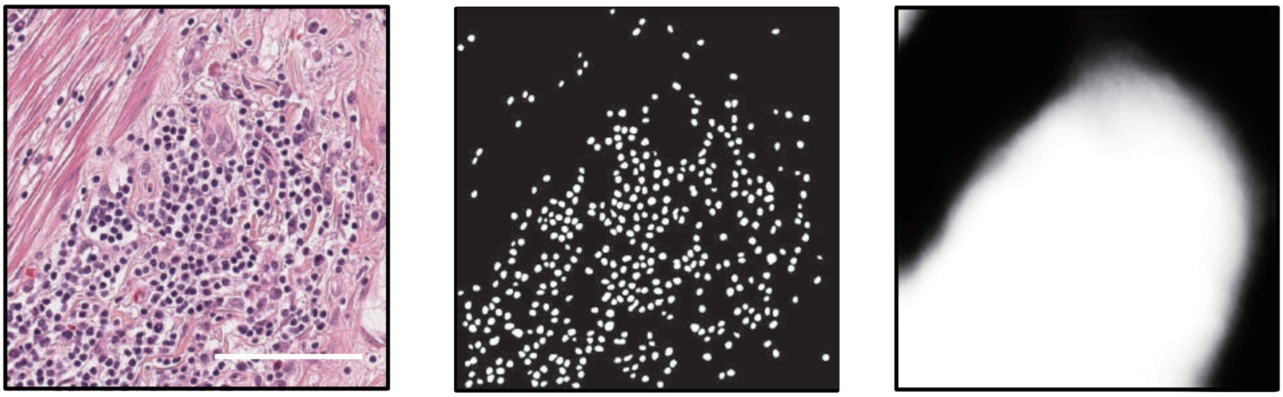

j

TCGA-SKCM

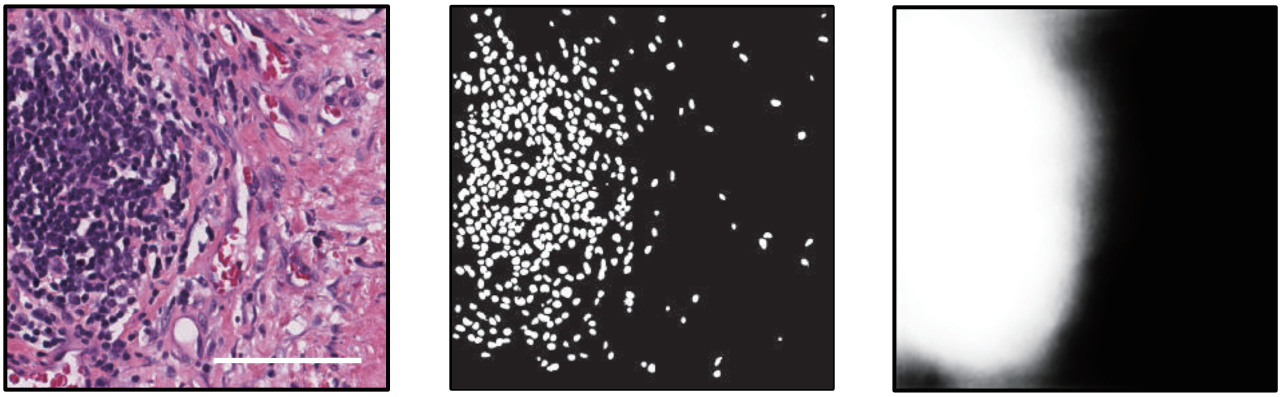

k

TCGA-PAAD

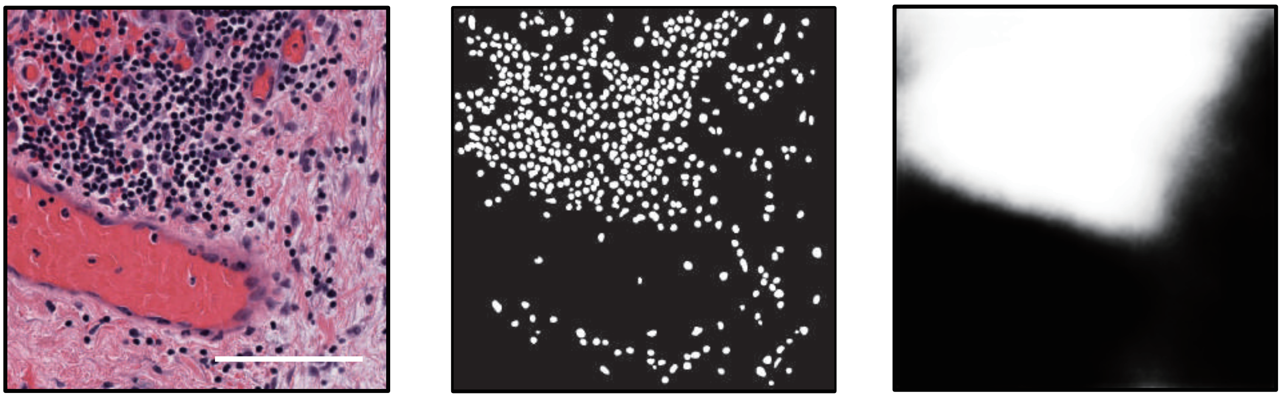

l

TCGA-CHOL

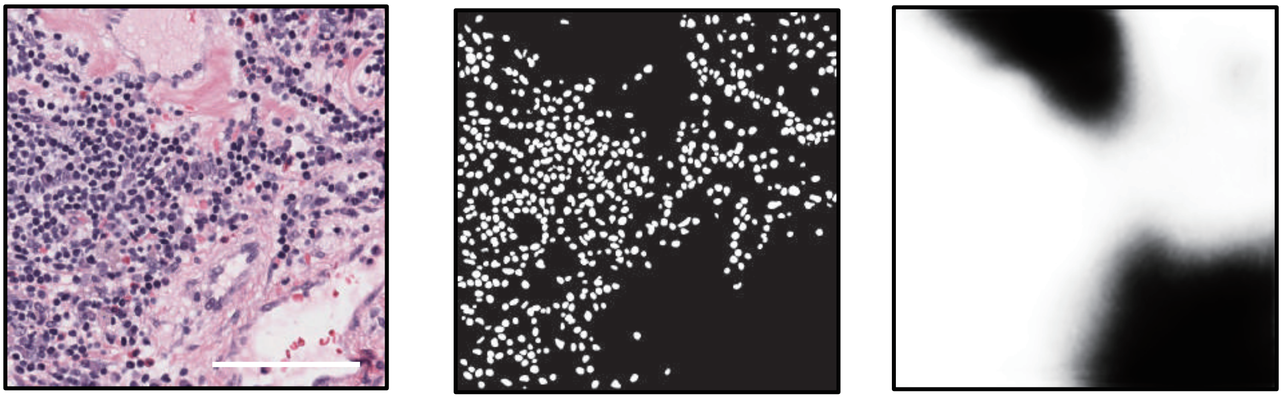

Supplementary Figure 6 (continued)

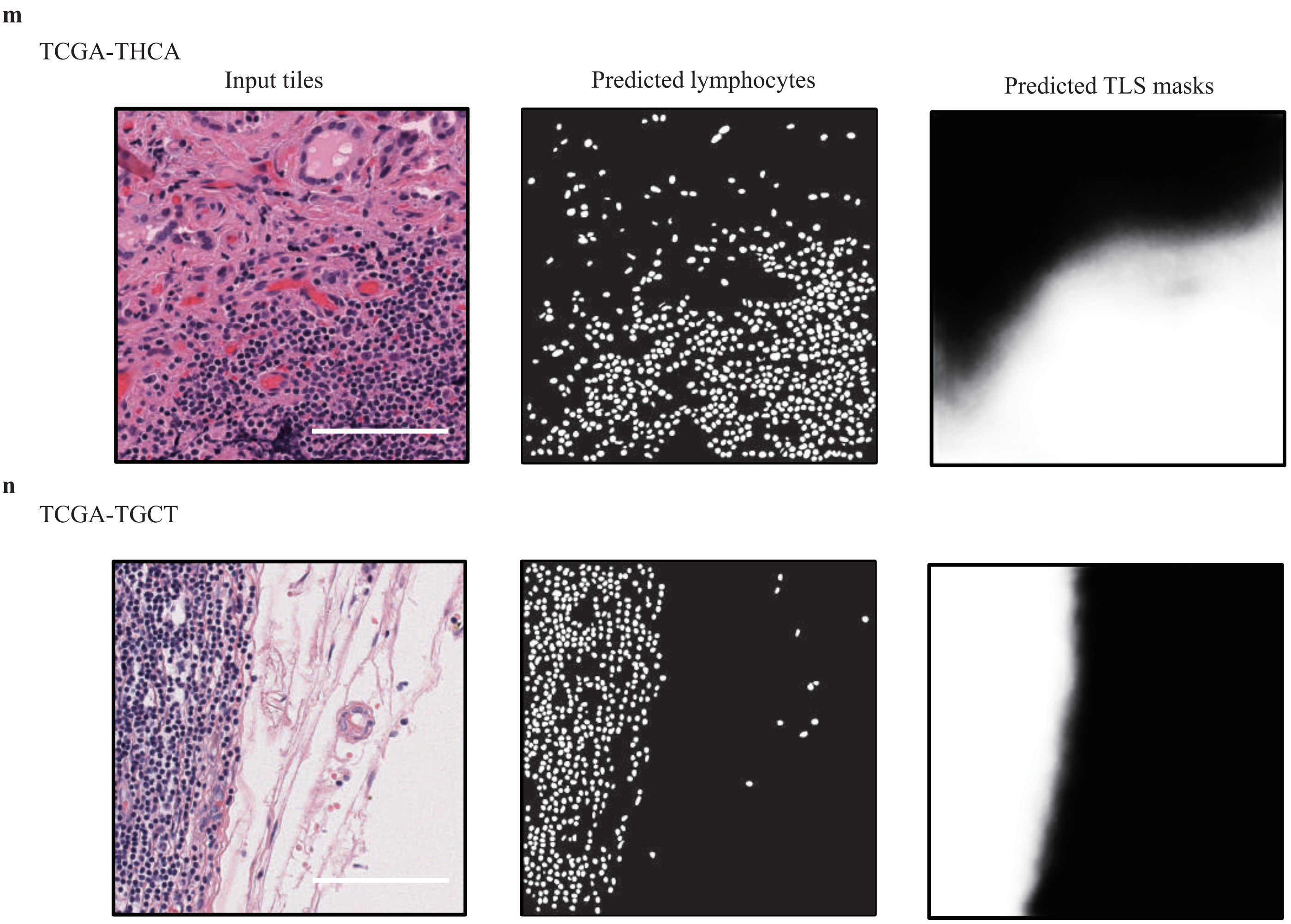

Supplementary Figure 7

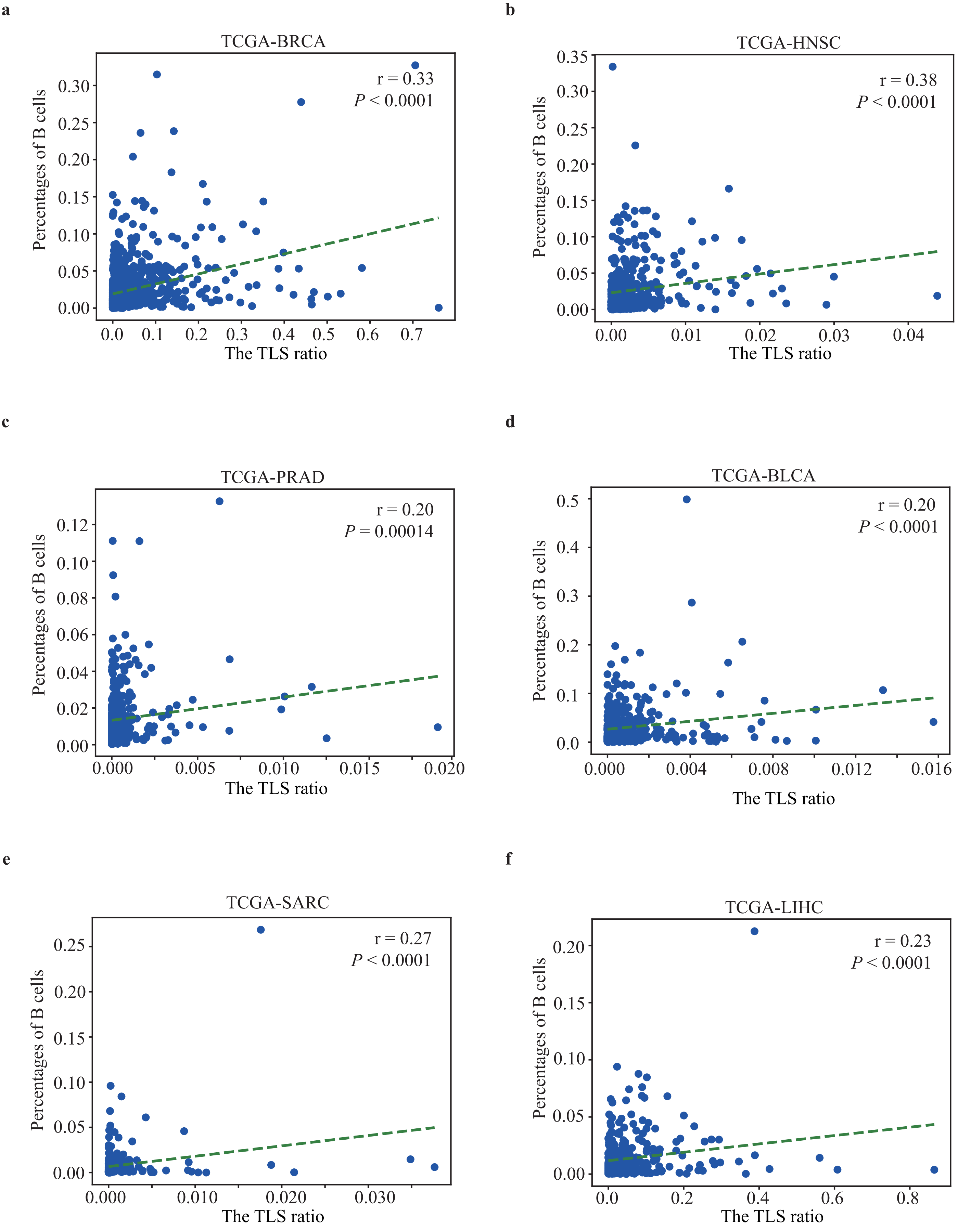

Supplementary Figure 7 (Continued)

g

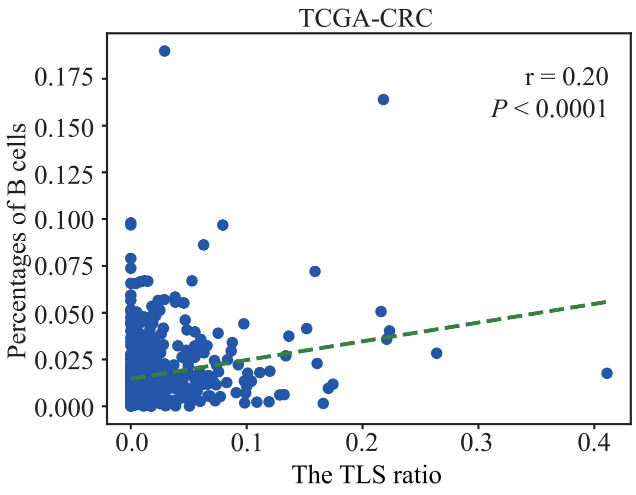

h

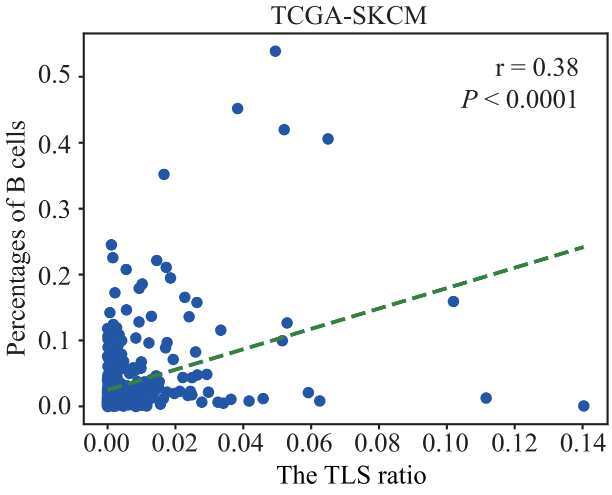

i

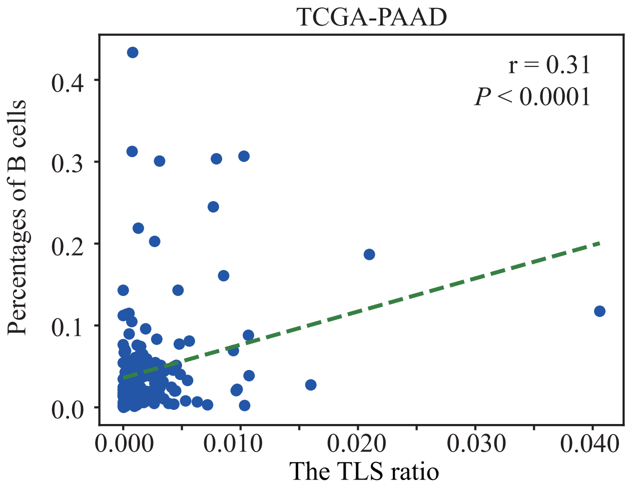

j

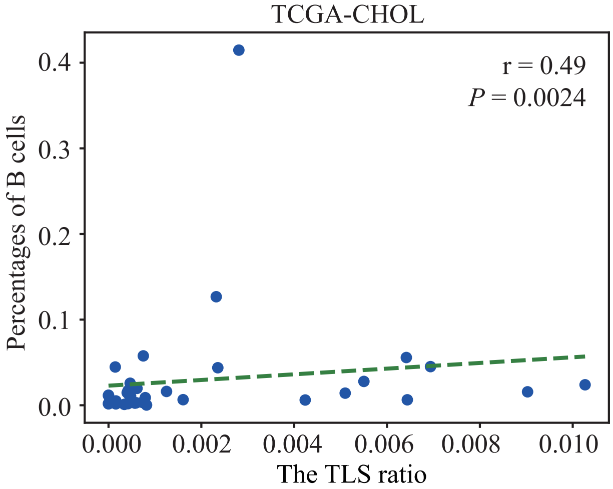

k

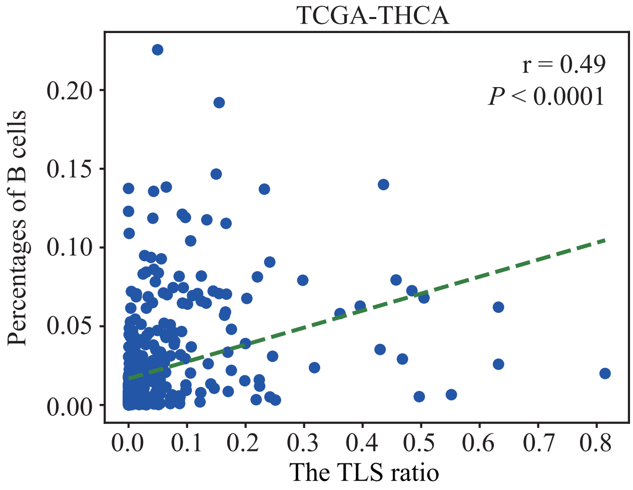

l

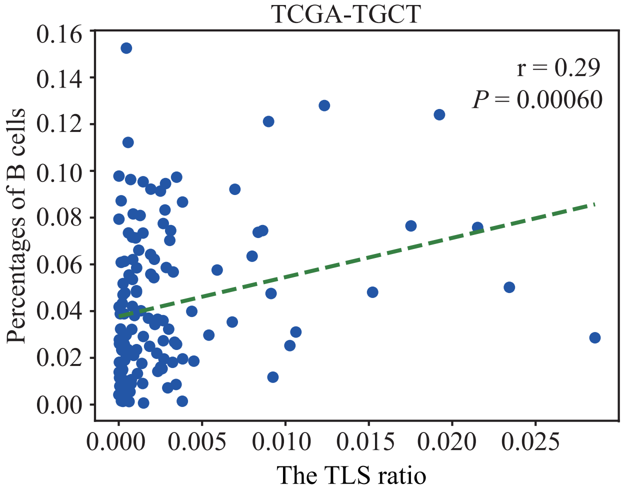

Supplementary Figure 8

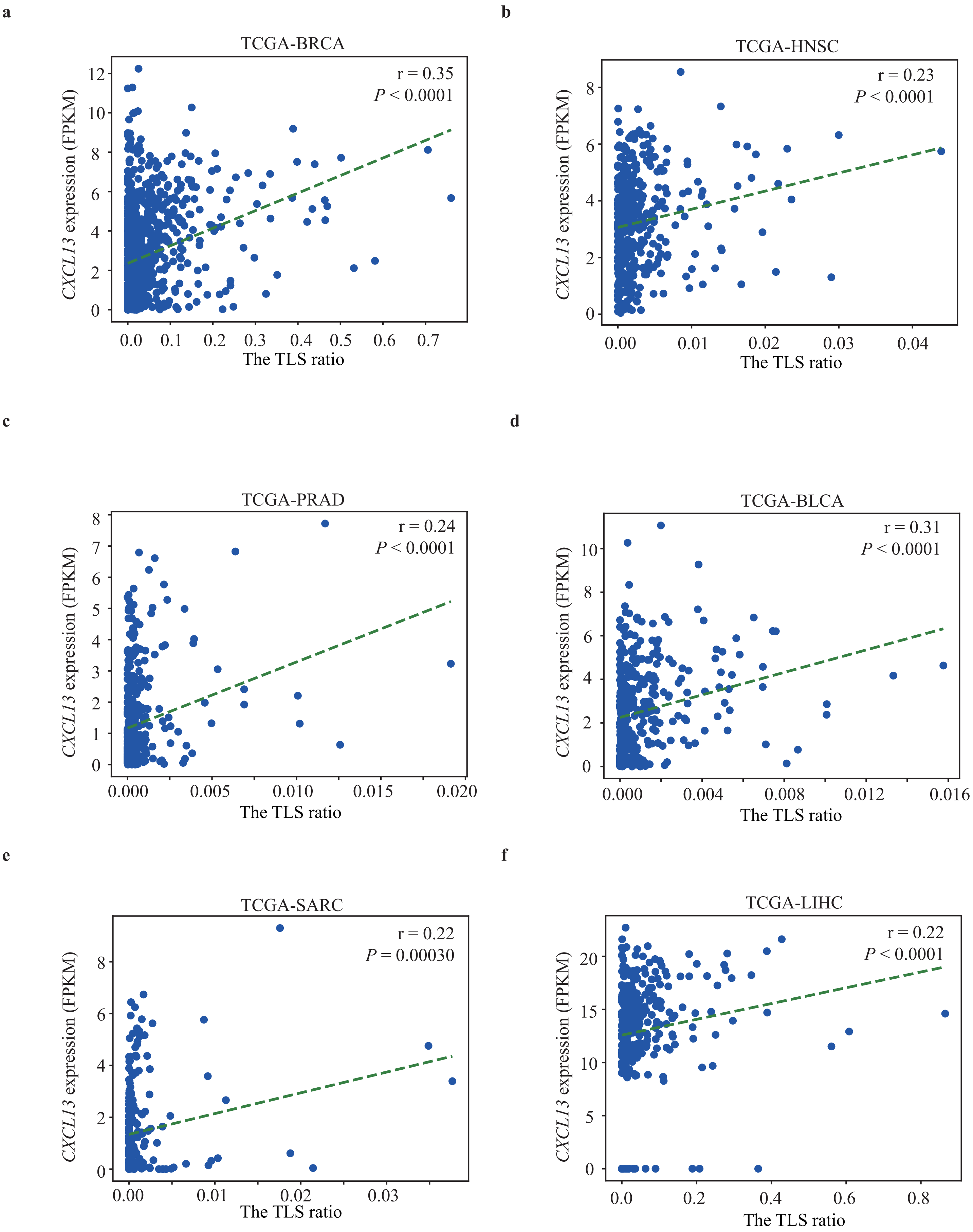

Supplementary Figure 8 (continued)

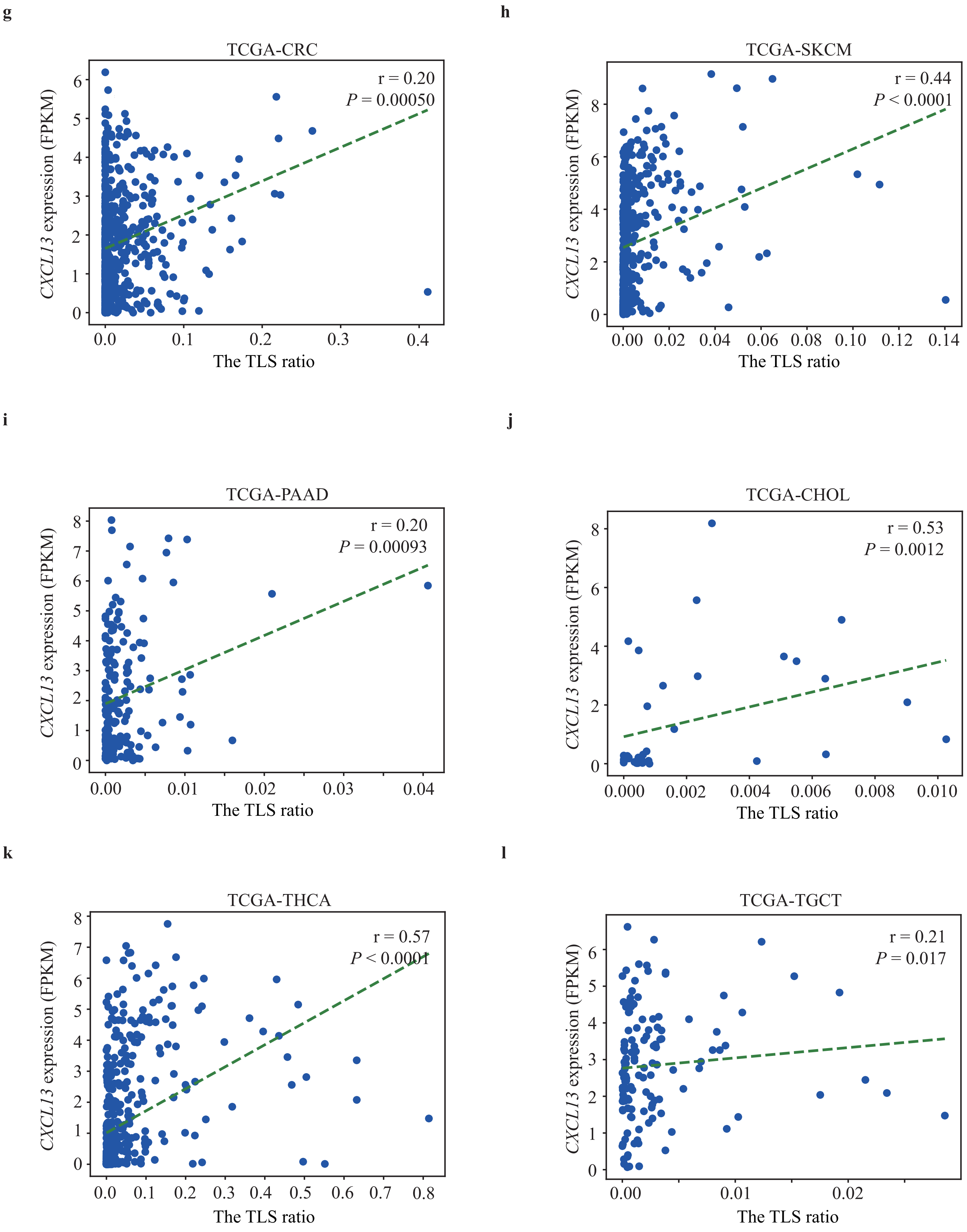

Supplementary Figure 9

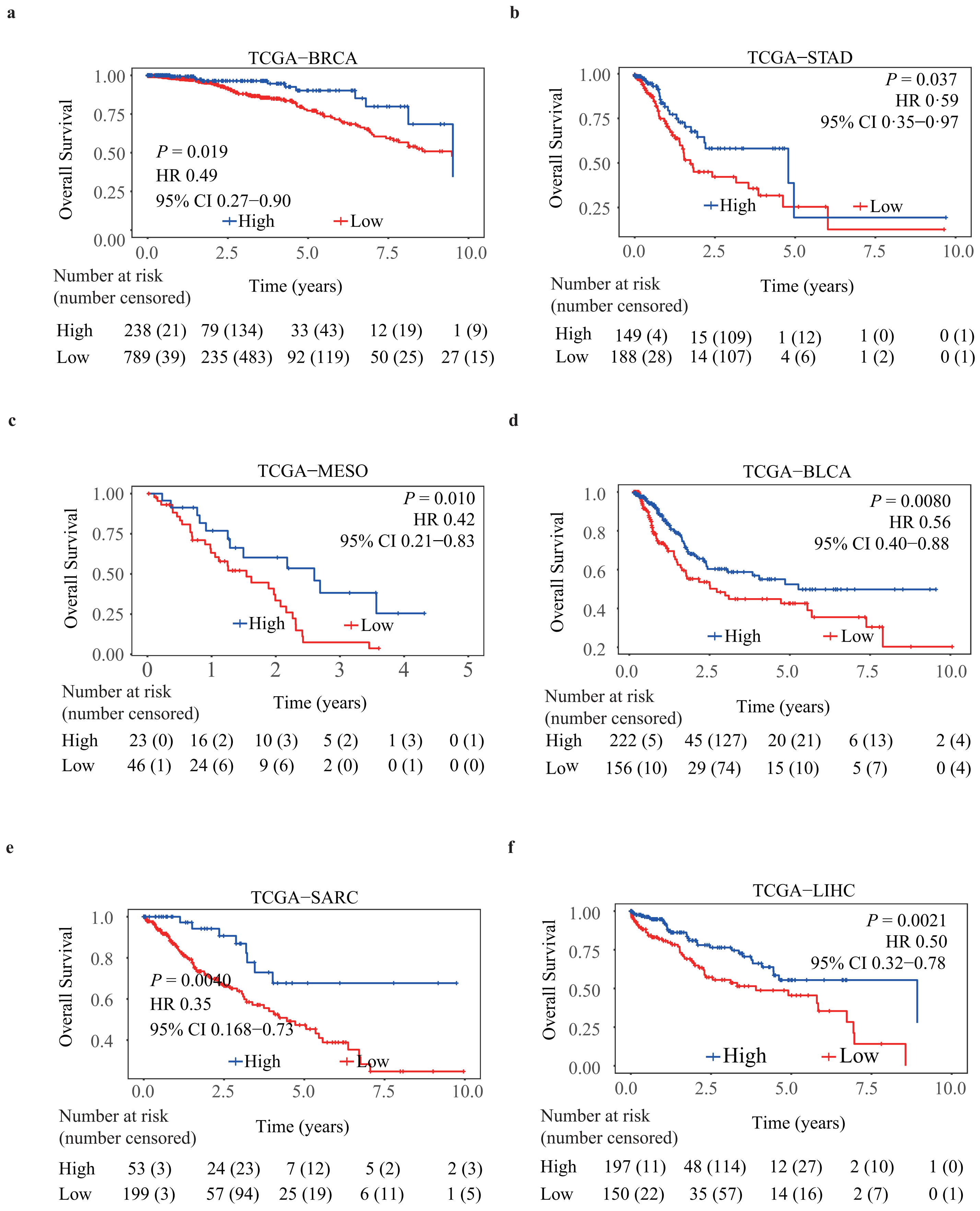

Supplementary Figure 9 (continued)

g

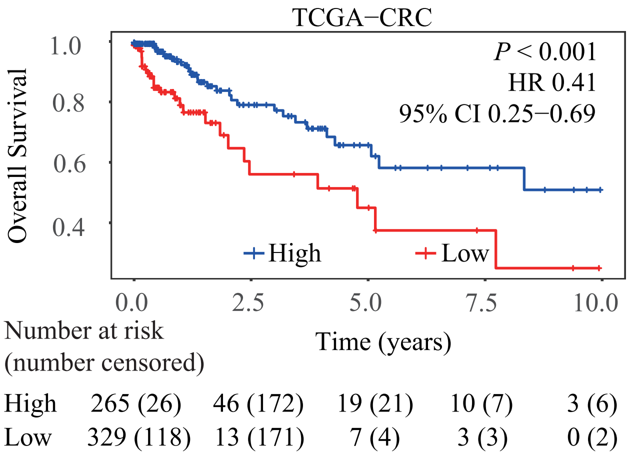

h

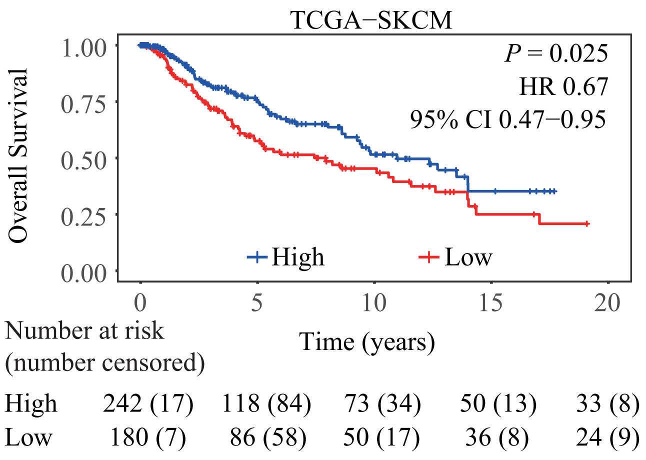

i

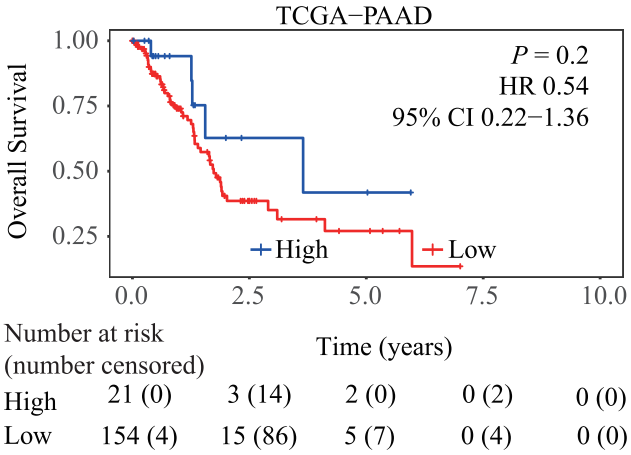

j

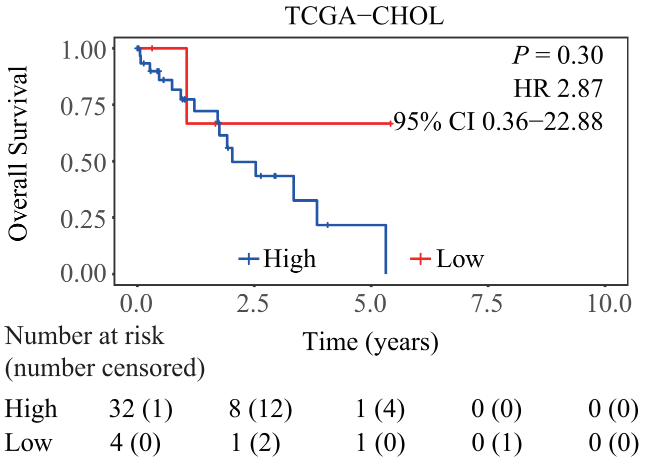

k

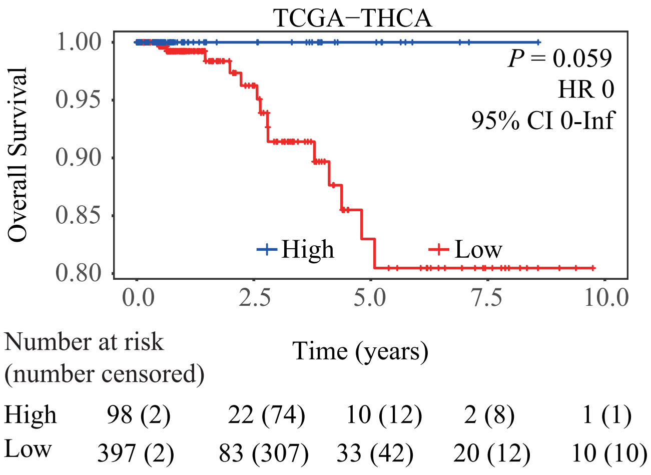

l

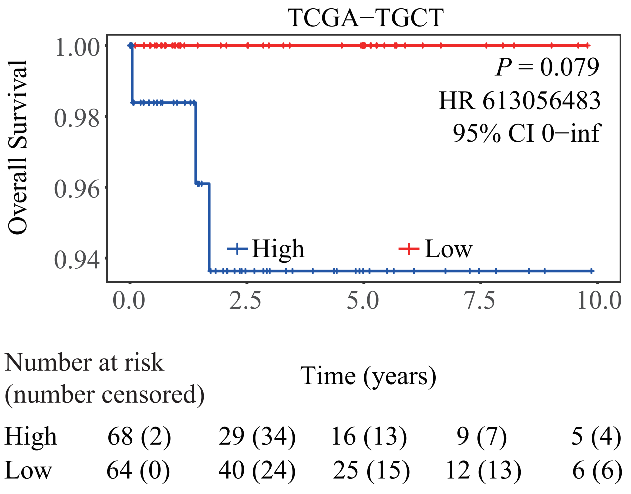

Supplementary Figure 9 (continued)

m

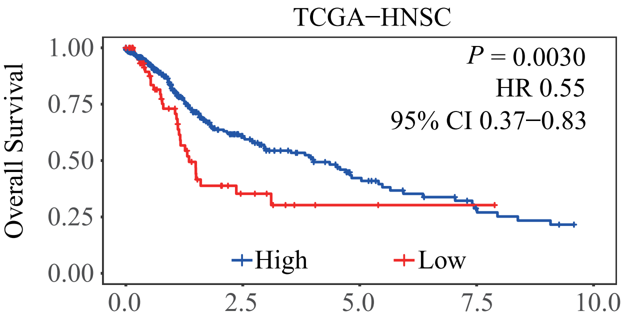

Number at risk
(number censored)

High	358 (4)	80 (187)	33 (28)	16 (8)	10 (2)
Low	64 (1)	9 (25)	2 (6)	1 (1)	0 (1)

n

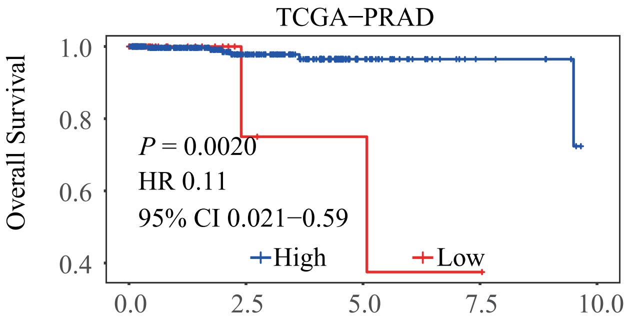

Number at risk
(number censored)

High	339 (0)	121 (214)	36 (84)	8 (28)	1 (6)
Low	60 (0)	3 (56)	2 (1)	1 (0)	0 (1)

Supplementary Figure 10

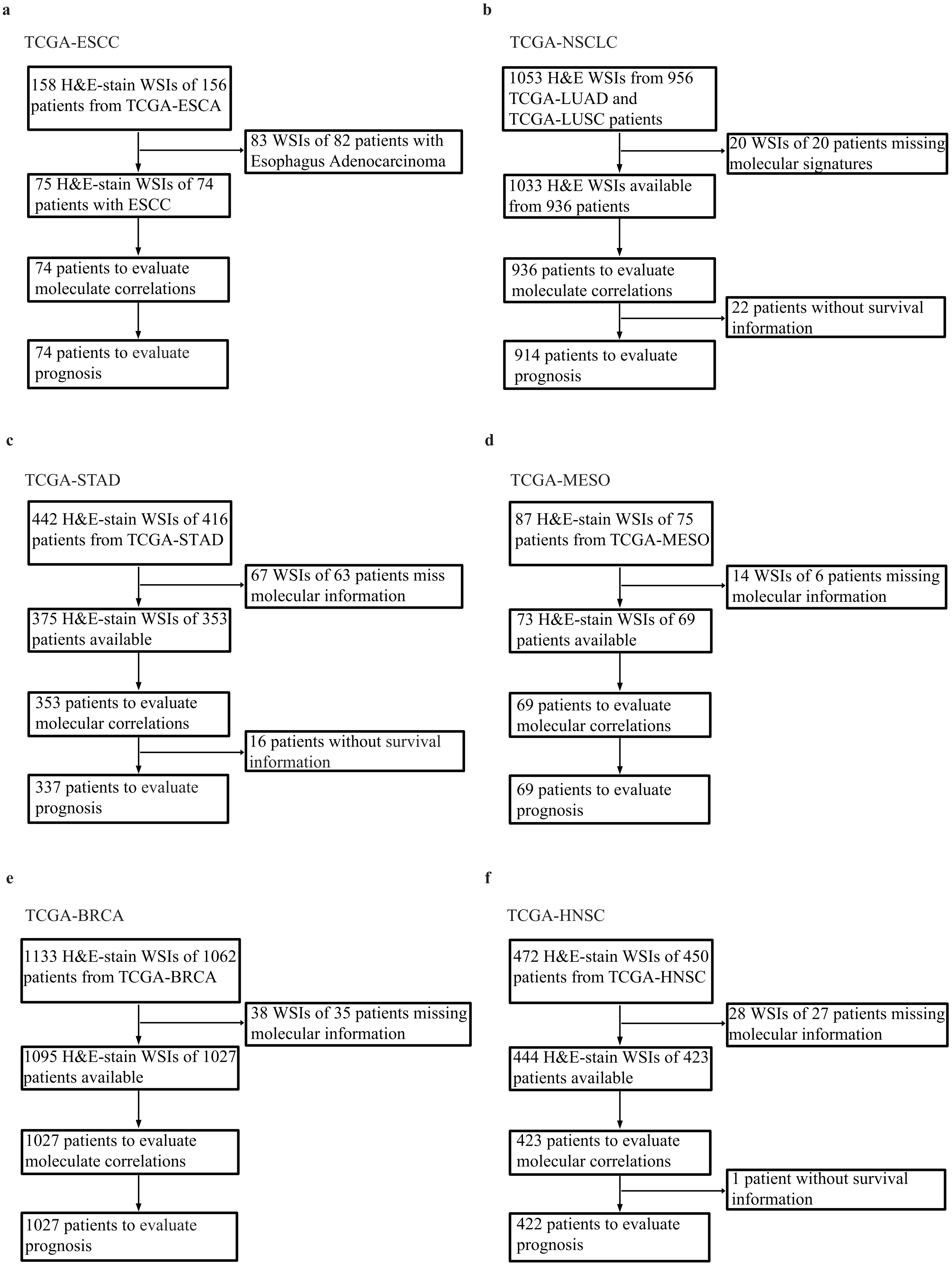

Supplementary Figure 10 (continued)

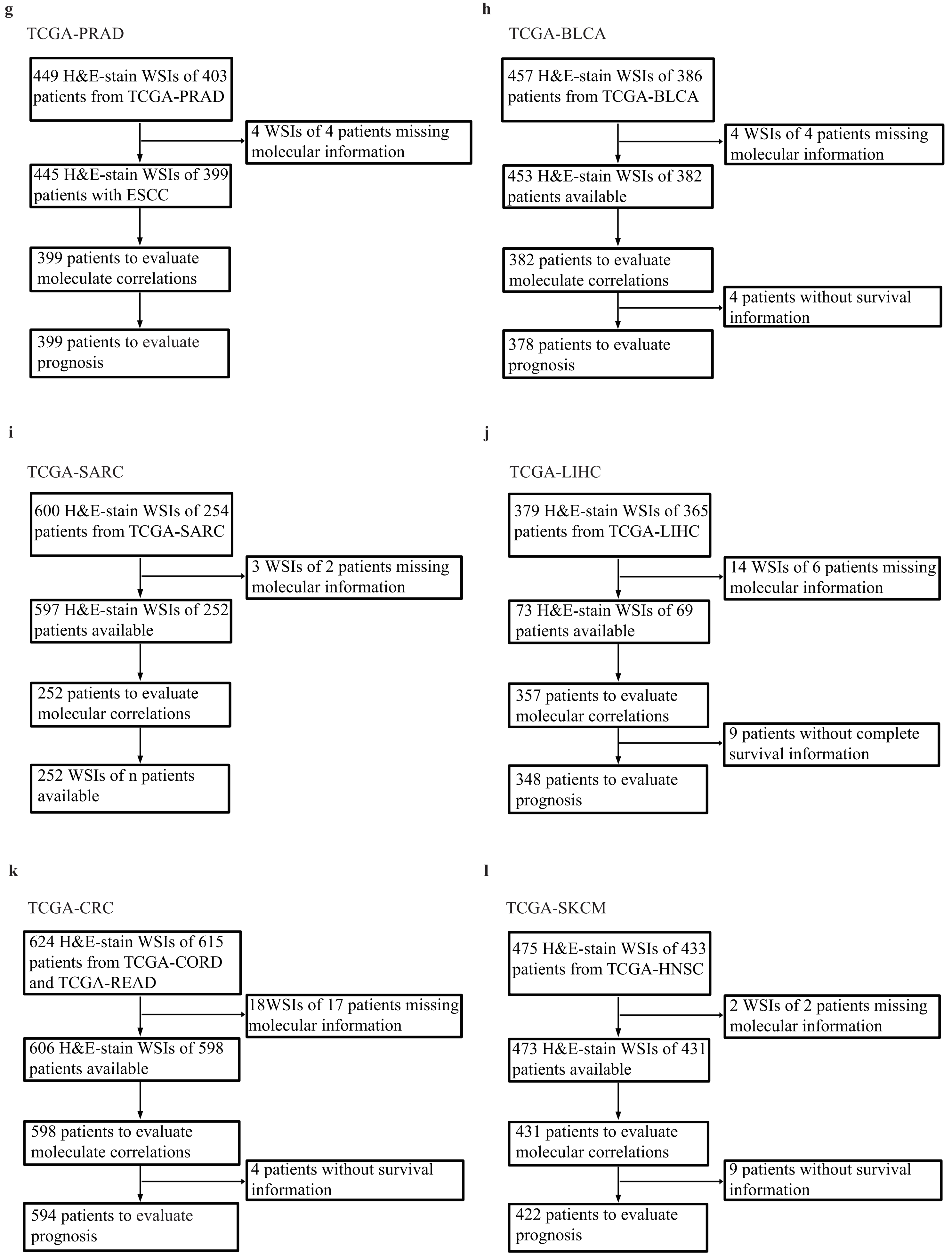

Supplementary Figure 10 (continued)

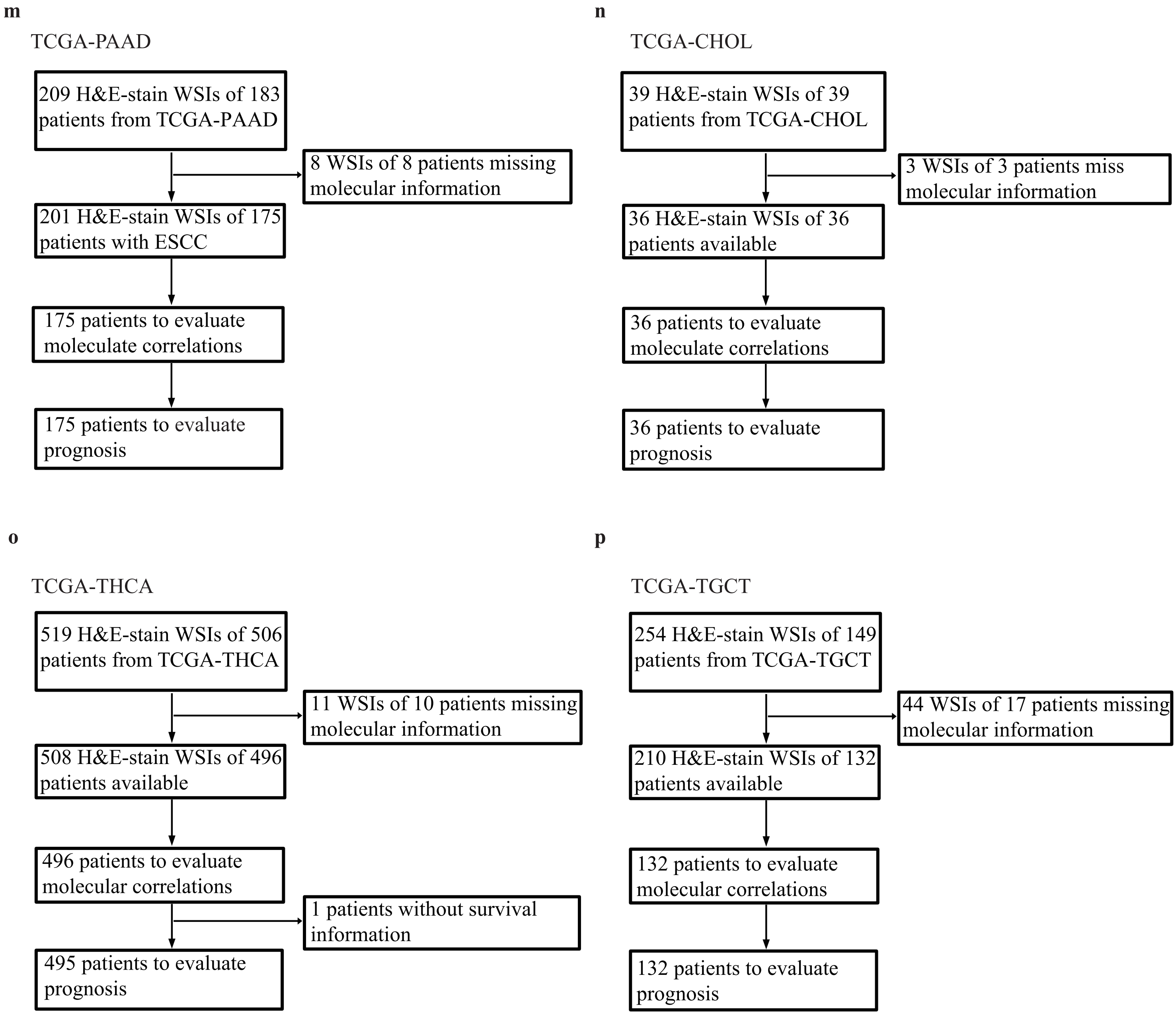

Supplementary Figure 11

a

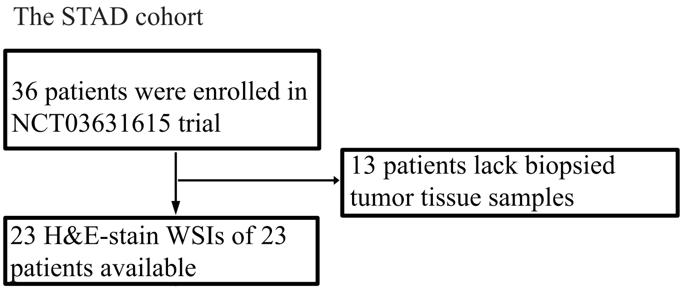

Supplementary Table 1 Clinical characteristics of ESCC and NSCLC patients used in the study

	Characteristics	Responder	Non-responder	*P* value
ESCC	Number	32	28	-
	Mean ages (±SD)	62.91 (±7.39)	61.18 (±8.50)	0.39 (Student's t test)
	Sex			0.62 (Fisher's exact test)
	Male	29	27	
	Female	3	1	
	TNM stage			-
	III	32	28	
NSCLC	Number	1	4	-
	Mean ages (±SD)	70 (n.a.)	64.75 (±5.26)	-
	Sex			0.25 (Fisher's exact test)
	Male	1	3	
	Female	0	1	
	TNM stage			-
	III	1	4	

Supplementary Table 2 Summary of various H&E staing conditions and scanners used in the internal training, validation and test sets

Tumor types	NSCLC		ESCC					
Various conditions	Condition 1		Condition 1		Condition 2		Condition 3	
Number of WSIs	5		60		17		14	
Number of tiles in the training set	760		8846		3314		2827	
Number of tiles in the validation set	116		1268		392		474	
Number of tiles in the test set	236		2471		968		825	
Performance metrics	IoU	Dice	IoU	Dice	IoU	Dice	IoU	Dice
	0.79<br>95% CI: 0.79-0.81	0.9<br>95% CI: 0.89-0.91	0.83<br>95% CI: 0.83-0.84	0.91<br>95% CI: 0.91-0.91	0.82<br>95% CI: 0.81-0.83	0.91<br>95% CI: 0.91-0.92	0.83<br>95% CI: 0.82-0.83	0.92<br>95% CI: 0.92-0.92

Condition 1: Hematoxylin stained for 5 minutes and Eosin stained for 2 minutes. Scanned with Perkin Elmer scanner.

Condition 2: Hematoxylin stained for 8 or 3 minutes and Eosin stained for 2 minutes twice or 1 minute. Scanned with Kfbio scanner.

Condition 3: Hematoxylin stained for 5 minutes and Eosin stained for 2 minute. Scanned with Olympus scanner.

Supplementary Table 3 Summary of four cohorts assessed for predicting immunotherapy responses

	Characteristics	Responder	Non-responder	*P* value
the ESCC cohort	Number	14	29	-
	Mean ages (±SD)	63.0 (±8.22)	59.5 (±6.07)	0.14 (Student's t test)
	Sex			0.82 (Fisher's exact test)
	Male	14	27	
	Female	0	2	
	Stage			-
	Stage I-III	0	0	
	Stage IV	14	29	
the NSCLC cohort receiving combined anti-PD-1 and chemotherapy	Number	28	28	-
	Mean ages (±SD)	63.35 (±7.56)	64.5 (±8.33)	0.73 (Student's t test)
	Sex			0.3 (Fisher's exact test)
	Male	25	21	
	Female	3	7	
	T stage			0.85 (Fisher's exact test)
	T1	8	6	
	T2	12	13	
	T3	5	5	
	T4	2	4	
	NA	1	0	
	N stage			0.31 (Fisher's exact test)
	N0	13	7	
	N1	2	4	
	N2	11	14	
	N3	1	3	
	NA	1	0	
	M stage			0.24 (Fisher's exact test)
	M0	25	28	
	M1	2	0	
	NA	1	0	
the NSCLC cohort receiving combined anti-PD-1 and apatinib therapy	Number	8	10	-
	Mean ages (±SD)	60.8 (±4.32)	56.1 (±9.55)	0.19 (Student's t test)
	Sex			
	Male	8	10	1 (Fisher's exact test)
	Female	0	0	
	T stage			0.44 (Fisher's exact test)
	T1	2	4	
	T2	6	4	
	T3	0	2	
	N stage			1 (Fisher's exact test)
	N0	1	1	
	N1	3	3	
	N2	4	6	
	M stage			1 (Fisher's exact test)
	M0	8	10	
	M1	0	0	
the STAD cohort	Number	14	9	-
	Mean ages (±SD)	63.35 (±7.56)	64.5 (±8.33)	0.73 (Student's t test)
	Sex			0.64 (Fisher's exact test)
	Male	11	6	
	Female	3	3	
	T stage			0.34 (Fisher's exact test)
	T3	2	3	
	T4a	12	6	

	N stage			1 (Fisher's exact test)
	N0	0	0	
	N+	14	9	
	M stage			1 (Fisher's exact test)
	M0	14	9	
	M1	0	0	

Supplementary Table 4 Univariate and multivariate survival analyses in the TCGA and CPTAC cohorts

	Univariate				Multivariate			
	Number	HR	95% CI	*P* value	Number	HR	95% CI	*P* value
TCGA-ESCC	74	0.28	0.09-0.85	**0.024**	66	0.14	0.03 - 0.72	**0.0183**
TCGA-NSCLC	914	0.74	0.57-0.95	**0.020**	906	0.79	0.61 - 1.03	0.0845
TCGA-MESO	69	0.42	0.21-0.83	**0.012**	69	0.55	0.25 - 1.19	0.1281
TCGA-STAD	337	0.59	0.35-0.97	**0.012**	290	0.62	0.34 - 1.12	0.1111
TCGA-BRCA	1027	0.49	0.27-0.90	**0.022**	1027	0.61	0.33 - 1.14	0.122
TCGA-HNSC	422	0.54	0.37-0.83	**0.0042**	422	0.6	0.39 - 0.94	**0.0239**
TCGA-PRAD	399	0.11	0.021-0.59	**0.0097**	307	0.11	0.02 - 0.61	**0.0122**
TCGA-BLCA	378	0.56	0.40-0.88	**0.0092**	348	0.69	0.42 - 1.13	0.137
TCGA-LIHC	348	0.5	0.32-0.78	**0.0024**	345	0.65	0.4 - 1.05	0.0785
TCGA-CRC	594	0.41	0.25-0.69	**0.0024**	556	0.44	0.25 - 0.78	**0.0047**
TCGA-SKCM	422	0.67	0.47-0.95	**0.025**	361	0.62	0.37 - 1.06	0.0799
TCGA-SARC	252	0.35	0.168-0.73	**0.0053**	NA	NA	NA	NA
TCGA-PAAD	175	0.54	0.22-1.36	0.19	173	0.43	0.18 - 1.05	0.0643
TCGA-CHOL	36	2.87	0.26-22.88	0.32	36	0.49	0.04 - 6.07	0.5763
TCGA-THCA	495	0	0 - Inf	1.00	494	0	0 - Inf	0.91
TCGA-TGCT	132	613056483	0 - Inf	1.00	117	0.62	0.37 - 1.06	0.0799
CPTAC-NSCLC	209	0.399	0.17-0.93	**0.034**	201	0.42	0.16 - 1.09	0.0729

Wald tests were used to calculate the *P* values for Harzard Ratios with either univariate or multivariate analyses. Significant *P* values less than 0.05 were highlighted in bold.

Supplementary Table 5 Comparison of C-Index results across various factors in the TCGA and CPTAC cohorts

	Number	C-Index			*P* value
		Factor 1	Factor 2	Factor 3	
TCGA-ESCC	66	0.689 (95% CI: 0.577-0.801)	0.738 (95% CI: 0.640-0.836)	**0.814 (95% CI: 0.688-0.940)**	0.008
TCGA-NSCLC	906	0.556 (95% CI: 0.521-0.591)	0.601 (95% CI: 0.558-0.644)	**0.614 (95% CI: 0.573-0.655)**	0.07
TCGA-MESO	69	0.564 (95% CI: 0.513-0.660)	0.717 (95% CI: 0.643-0.791)	**0.722 (95% CI: 0.649-0.797)**	0.07
TCGA-STAD	290	0.546 (95% CI: 0.515-0.577)	0.611 (95% CI: 0.528-0.703)	**0.648 (95% CI: 0.558-0.738)**	0.03
TCGA-BRCA	1027	0.568 (95% CI: 0.528-0.607)	0.723 (95% CI: 0.658-0.788)	**0.745 (95% CI: 0.688-0.802)**	0.04
TCGA-HNSC	422	0.548 (95% CI: 0.511-0.585)	0.571 (95% CI: 0.520-0.622)	**0.607 (95% CI:0.567-0.647)**	0.02
TCGA-PRAD	307	0.591 (95% CI: 0.532-0.650)	0.658 (95% CI:0.63-0.684)	**0.712 (95% CI: 0.685-0.739)**	0.034
TCGA-BLCA	348	0.563 (95% CI: 0.520-0.614)	0.675 (95% CI: 0.612-0.738)	**0.687 (95% CI: 0.622-0.752)**	0.075
TCGA-LIHC	345	0.597 (95% CI: 0.540-0.662)	0.603 (95% CI: 0.530-0.678)	**0.654 (95% CI: 0.586-0.724)**	0.027
TCGA-CRC	556	0.646 (95% CI: 0.566-0.726)	0.681 (95% CI: 0.595-0.767)	**0.738 (95% CI: 0.675-0.809)**	0.002
TCGA-SKCM	361	0.547 (95% 0.510-0.584)	0.681 (95% CI: 0.628-0.734)	**0.687 (95% CI: 0.634-0.740)**	0.048
TCGA-PAAD	173	0.540 (95% CI: 0.510-0.570)	0.60 (95% CI: 0.526-0.674)	**0.645 (95% CI: 0.572-0.718)**	0.029
TCGA-CHOL	36	0.53 (95% CI: 0.523-0.538)	0.762 (95% CI: 0.600-0.920)	**0.769 (95% CI: 0.604-0.934)**	0.67
TCGA-TGCT	117	0.768 (95% CI: 0.711-0.825)	0.81 (95% CI: 0.643-0.987)	**0.891 (95% CI: 0.795-0.987)**	0.15
TCGA-THCA	494	0.596 (95% CI: 0.566-0.627)	0.743 (95% CI: 0.594-0.891)	**0.763 (95% CI: 0.658-0.901)**	0.020
CPTAC-NSCLC	201	0.57 (95% CI: 0.533-0.604)	0.673 (95% CI: 0.602-0.744)	**0.701 (95% CI: 0.636-0.766)**	0.045

Factor 1: TLS; Factor 2: T + N + M; Factor 3: T + N + M + TLS

A Likelihood ratio test was used to calculate the *P* value comparing the nested Cox regression model between Factor 3 (T + N + M + TLS ratio) and Factor 2 (T + N + M) across various tumor types

C-Indexes with best discriminative power were highlighted in bold.
